# Chidamide and cytarabine synergistically treat acute myeloid leukemia: inhibiting ribosome biogenesis via the MYC-RRP9 pathway

**DOI:** 10.1038/s41419-025-07928-y

**Published:** 2025-08-09

**Authors:** Qing Li, Fangfang Wang, Xinrong Xiang, Lei Zhao, Xuefeng Li, Ying Qu, Jingcao Huang, Yunfan Yang, Yang Dai, Xiao Shuai, Jiazhuo Liu, Jie Huang, Yu Liu, Yu Wu

**Affiliations:** 1 Department of Hematology and Institute of Hematology, West China Hospital, Sichuan University, Chengdu, Sichuan China; 2 Department of Hematology and Institute of Hematology, State Key Laboratory of Biotherapy and Cancer Center, West China Hospital, Sichuan University, Chengdu, Sichuan China; 3 Department of Hematology and Institute of Hematology, State Key Laboratory of Biotherapy, West China Hospital, Sichuan University, Chengdu, Sichuan China

**Keywords:** Acute myeloid leukaemia, Acute myeloid leukaemia

## Abstract

This study explores innovative therapeutic approaches for acute myeloid leukemia by examining the synergistic effects of the histone deacetylase inhibitor chidamide in combination with cytarabine. In both in vitro and in vivo models, the drug combination demonstrated significant synergism in combating acute myeloid leukemia. Transcriptomic analysis revealed that the combination treatment notably downregulates the MYC signaling pathway. Binding assays, including surface plasmon resonance, molecular docking, and molecular dynamics simulations, further demonstrated that chidamide directly interacts with the C-MYC protein, while cytarabine enhanced this interaction. Delving deeper into the mechanism, we identified *RRP9*—an essential gene for ribosome biogenesis and a key player in acute myeloid leukemia prognosis—as a critical component of the MYC pathway. It was confirmed that MYC regulates RRP9 expression. Functional assays showed that overexpression of RRP9 promoted acute myeloid leukemia cell proliferation and resistance to the chidamide-cytarabine combination, whereas RRP9 knockdown impaired rRNA synthesis, reduced nucleolar size, and diminished protein production. Ultimately, we found that chidamide combined with cytarabine effectively inhibit ribosome biogenesis in acute myeloid leukemia cells. These results underscore the therapeutic potential of targeting the MYC-RRP9 axis to disrupt ribosome biogenesis in acute myeloid leukemia, offering a promising avenue for acute myeloid leukemia treatment.

## Introduction

Acute myeloid leukemia (AML) is a heterogeneous malignancy marked by the uncontrolled proliferation of clonal hematopoietic cells [1, 2]. The overall 5-year survival rates in patients with AML were 28% in the decades 2010 to 2017 [1]. Since 2017, twelve new drugs, including targeted therapies such as venetoclax, FLT3 inhibitors, IDH inhibitors, and epigenetic modifiers, have been approved for AML treatment [3]. Increasing research is focusing on integrating these targeted therapies with the conventional “3 + 7” chemotherapy regimen [4, 5]. These innovations have enabled personalized and targeted treatment in AML, improving patient outcomes and quality of life [6]. Nevertheless, the overall survival rate remains stagnant, underscoring the pressing need for innovative therapeutic strategies.

Chidamide (CS055), a novel oral histone deacetylase (HDAC) inhibitor targeting HDAC1, 2, 3, and 10, induces tumor cell cycle arrest, promotes apoptosis, and enhances antitumor immunity. Approved by the Chinese FDA in 2014 for peripheral T-cell lymphoma [7], it received expanded approval in 2024 for treating newly diagnosed diffuse large B-cell lymphoma with dual MYC and BCL2 expression. Chidamide, when combined with AML treatments like daunorubicin, decitabine, or the CAG regimen (cytarabine, aclarubicin, and granulocyte colony stimulating factor), has shown significant therapeutic potential [8–10]. Clinical trials have reported a complete remission rate of 45% with the chidamide and CAG regimen in patients with relapsed AML/MDS [11]. Another multicenter study confirmed an overall response rate of 46.2% when chidamide was paired with epigenetic modifiers and standard chemotherapy [12]. These findings underline its promising efficacy in AML therapy, though mechanisms remain unclear.

Dysregulated ribosome biogenesis, a hallmark of various cancers including AML, fuels unchecked cellular growth and proliferation, ultimately driving disease progression and correlating with unfavorable patient outcomes [13–17]. Ribosome biogenesis is a multi-stage process beginning in the nucleolus and culminating in ribosome assembly. Nucleolar shape, size, and number correlate with ribosome production efficiency and the cell’s metabolic and proliferation status [18]. Nucleophosmin 1 (NPM1), located in the nucleolus, is involved in ribosome biogenesis [19]. Ribosomal DNA is transcribed into 47S pre-rRNA, which is processed into 5.8S, 18S, and 28S rRNAs [20]. RRP9, a subunit of the U3 small nucleolar ribonucleoproteins (snoRNPs), is essential for early pre-rRNA cleavage at the A0, A1, and A2 sites [21, 22]. Database analyses show a strong correlation between elevated RRP9 expression and poorer AML prognosis [23]. However, its regulatory mechanisms and precise role in AML progression remain largely unexplored.

Preclinical studies suggest that targeting ribosome biogenesis or protein synthesis shows promising efficacy in AML treatment [24, 25]. CX-5461, a selective ribosomal DNA transcription inhibitor, has demonstrated antitumor activity and long-term safety in hematological malignancies [26]. MYC, a key regulator of ribosome biogenesis and protein synthesis, is a well-established proto-oncogene involved in production of ribosomal components [27, 28]. Furthermore, studies have demonstrated that MYC enhances the survival of leukemia stem cells [29]. HDAC inhibitors have shown promise in targeting MYC-driven cancers [30]. This study aims to assess the therapeutic effects of HDAC inhibitors chidamide combined with cytarabine in AML, focusing on their ability to inhibit ribosome biogenesis via the MYC pathway. This approach seeks to provide new insights and potential targets for AML treatment.

## Results

### Synergistic therapeutic effects of chidamide and cytarabine in AML cells

**Fig. 1:**
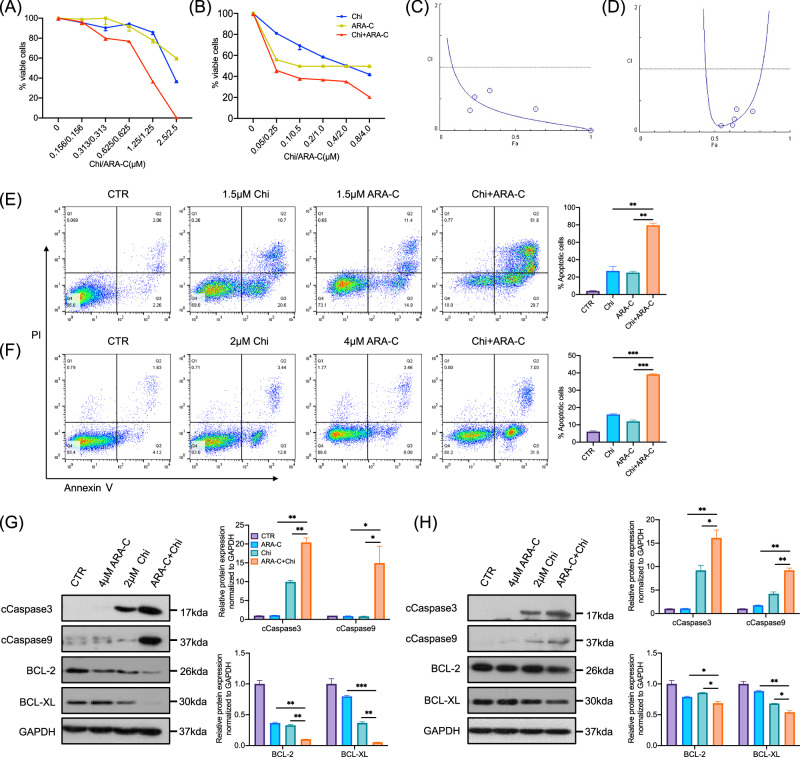
Synergistic therapeutic effects of chidamide and cytarabine in AML cells. **A**, **B** Cytotoxic effects of chidamide and cytarabine, alone or in combination, on MV4-11 cells (left) and Kasumi-1 cells (right). **C**, **D** Combination index plot, the horizontal axis (Fa) represents the inhibition rate, while the vertical axis shows CI values. **E**, **F** In MV4-11 cells, significant differences were observed between chidamide and combination (*P* = 0.0055) and between cytarabine and combination (*P* = 0.0014). In Kasumi-1 cells, both comparisons were also significant (chidamide vs. combination: *P* = 0.0002; cytarabine vs. combination: *P* = 0.0005; unpaired t-test). **G**, **H** Western blot analysis of cleaved caspase-3, cleaved caspase-9, BCL-2, and BCL-XL in MV4-11 (left) and Kasumi-1 (right) cells. For MV4-11: ARA-C vs. combination (*P* = 0.0019, 0.0468, 0.0013, 0.0003); chidamide vs. combination (*P* = 0.0071, 0.0463, 0.0013, 0.0018). For Kasumi-1: ARA-C vs. combination (*P* = 0.0063, 0.0021, 0.0420, 0.0027); chidamide vs. combination (*P* = 0.0397, 0.0077, 0.0140, 0.0120) (unpaired t-test). CTR (control), Chi (chidamide), ARA-C (cytarabine), Chi+ARA-C (chidamide + cytarabine); cCASP3, cleaved Caspase-3; cCASP9, cleaved Caspase-9. *P < 0.05, **P < 0.01, ***P < 0.001.

We assessed the toxicity of chidamide and cytarabine on MV4-11 and Kasumi-1 cells using the MTT assay. Both chidamide and cytarabine inhibited the growth of AML cell lines in a dose- and time-dependent manner (Fig. S1A–L). The half-maximal inhibitory concentrations (IC50) of chidamide and cytarabine at 24, 48, and 72 hours are presented in Table S1. Notably, the combination of chidamide and cytarabine induced greater cell death in MV4-11 and Kasumi-1 cells compared to either drug alone (Fig. 1A, B), and a similar effect was observed in HL60 cells (Fig. S2A). Combination index (CI) values were calculated using CompuSyn based on the Chou-Talalay method to assess drug interaction. A CI < 1 indicates synergy, =1 additive effect, and >1 antagonism. In MV4-11 and Kasumi-1 cells, all CI values were <1, indicating synergistic effects between chidamide and cytarabine (Fig. 1C, D). Synergistic effects were also observed in HL60 cells (Fig. S2B).

We employed flow cytometry to assess apoptosis induction in AML cell lines treated with the combination of chidamide and cytarabine. Compared to cytarabine monotherapy, the percentage of apoptotic MV4-11 cells increased from 25.11% ± 1.69% to 79.65% ± 2.33% (Fig. 1E), and that of Kasumi-1 cells rose from 11.99% ± 0.75% to 39.18% ± 0.35% (Fig. 1F) after 48 hours of co-treatment. In HL60 cells, apoptosis increased from 2.98% ± 0.24% to 39.45% ± 0.92% (Fig. S2C).

Western blot analysis revealed that the chidamide-cytarabine combination significantly upregulated the expression of cleaved Caspase-3 and cleaved Caspase-9, while downregulating BCL-2 and BCL-XL in both MV4-11 cells (Fig. 1G) and Kasumi-1 cells (Fig. 1H). Similar changes were also observed in HL60 cells (Fig. S2D). Furthermore, western blot showed that the combination treatment significantly enhanced histone acetylation, increasing levels of ace-H3K9, ace-H3K18, and ace-H4K16 in both MV4-11 cells (Fig. S3A) and Kasumi-1 cells (Fig. S3B).

### Synergistic therapeutic effects of chidamide and cytarabine in AML in vivo

**Fig. 2:**
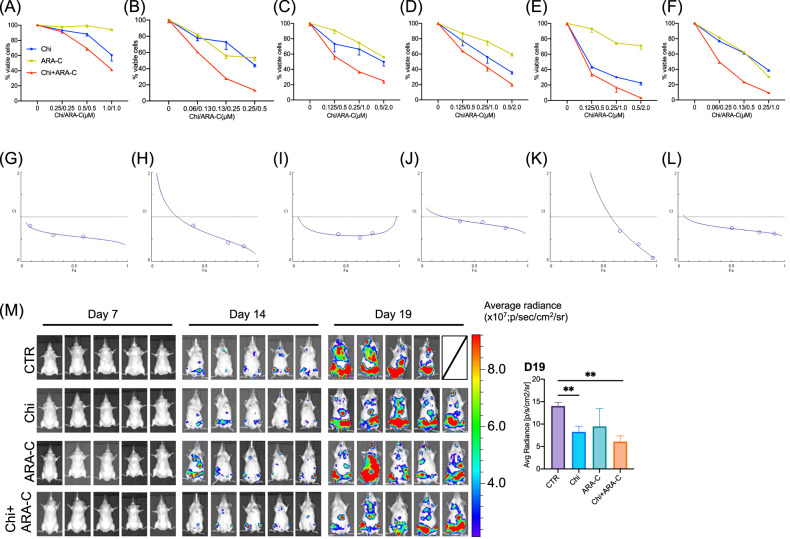
Synergistic therapeutic effects of chidamide and cytarabine in AML in vivo. **A**–**F** Cytotoxic effects of chidamide and cytarabine alone or in combination on PBMCs from 6 AML patients. **G**–**L** Combination index plot of 6 patients, the horizontal axis (Fa) represents the inhibition rate, while the vertical axis shows CI values. **M** In vivo images of mice on days 7, 14, and 19 after treatment with control, chidamide, cytarabine, or their combination. Fluorescence intensities were quantified using Living Image software. Statistical analysis (unpaired t-test) with GraphPad Prism 8.0 showed significant differences between control and chidamide or chidamide + cytarabine groups (*P* = 0.0086 and 0.0020, respectively) (*n*  = 5). CTR (control), Chi (chidamide), ARA-C (cytarabine), Chi+ARA-C (chidamide+ cytarabine). **P < 0.01.

We employed the CCK-8 assay to evaluate the cytotoxic effect of chidamide combined with cytarabine on primary AML cells. The combination treatment demonstrated a stronger inhibition on primary AML cells from six patients compared to either drug alone (Fig. 2A–F). Across the tested concentration ranges, the CI remained below 1 (Fig. 2G–L), confirming the synergistic cytotoxicity of chidamide and cytarabine. Clinical details of the six AML patients are provided in Table S2. We established an AML xenograft mouse model by intravenously injecting immunodeficient mice with MV4-11-luciferase-GFP cells, which were generated by infecting MV4-11 cells with lentivirus carrying both luciferase and GFP, to investigate the in vivo synergistic therapeutic potential of the drug combination. Over the treatment period, the fluorescence intensity difference between treatment groups became increasingly pronounced. Chidamide monotherapy significantly reduced tumor burden, while its combination with cytarabine achieved an even greater reduction (Fig. 2M).

In clinical practice, we registered the clinical trial of chidamide for the treatment of AML with the Chinese Clinical Trial Registry (https://www.chictr.org.cn/) (registration number ChiCTR2300073732), and five AML patients previously enrolled received chidamide-based treatment (20 mg twice a week). Notably, this regimen achieved complete remission (CR) in both high-risk patients at diagnosis (patients 1 and 2) and relapsed patients (patients 3 and 4). In addition, in a patient with persistent minimal residual disease (MRD) positivity (patient 5), MRD was successfully converted to negative for 13 months. Detailed patient data are shown in Table S3. Enrollment in this clinical trial is ongoing.

### Chidamide combined with cytarabine synergistically suppresses the MYC signaling pathway

**Fig. 3:**
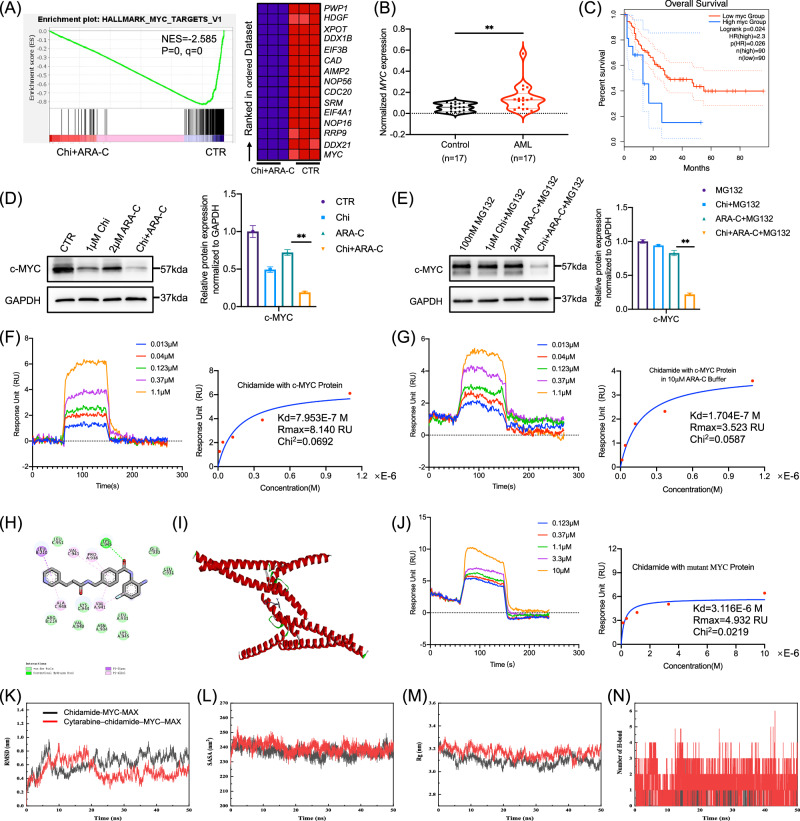
Chidamide combined with cytarabine synergistically suppresses the MYC signaling pathway. **A** Compared with the control group, the MYC pathway was significantly downregulated in the chidamide combined with cytarabine group. **B** *MYC* mRNA levels in PBMCs from AML patients and healthy individuals (*P* = 0.0089, unpaired t-test) (*n* = 17). **C** Data from the GEPIA2 website showing that high MYC expression in AML patients is significantly associated with poor prognosis (*P* = 0.0260, Kaplan Meier analysis), cutoff-high (15%), cutoff-low (85%). **D** Effects of chidamide and cytarabine on c-MYC protein levels in MV4-11 cells, with quantification of the results. Significant difference between cytarabine and combination (*P* = 0.0031, unpaired t-test). **E** c-MYC protein levels in MV4-11 cells were evaluated and quantified after treatment with chidamide and cytarabine in the presence of MG132. Significant difference between cytarabine and combination (*P* = 0.0026, unpaired t-test). **F** SPR assay demonstrating chidamide’s affinity for c-MYC recombinant protein. **G** SPR assay showing the effect of 10 µM cytarabine on chidamide’s affinity for c-MYC recombinant protein. **H**, **I** Two-dimensional (left) and three-dimensional (right) representations of molecular docking between chidamide and the MYC-MAX complex. **J** SPR analysis showing binding affinity of chidamide to MYC protein carrying point mutations at the predicted binding sites. **K–N** Simulation of chidamide binding to the MYC protein, with or without cytarabine, illustrating RMSD, SASA, Rg, and hydrogen bond (from left to right) dynamics over time. CTR (control), Chi (chidamide), ARA-C (cytarabine), Chi+ARA-C (chidamide + cytarabine). **P < 0.01.

To investigate the synergistic mechanisms of chidamide and cytarabine in AML, transcriptome sequencing (RNA-seq) was performed on MV4-11 cells, a human AML cell line harboring FLT3-ITD mutation and t(4;11) translocation. The combination treatment significantly downregulated the MYC signaling pathway (Fig. 3A). Notably, this inhibition was more pronounced with the combination treatment than with either drug alone (Fig. S4A, B). We further quantified MYC mRNA levels by quantitative real-time PCR (qPCR) in peripheral blood mononuclear cells (PBMCs) from 17 AML patients and 17 healthy controls, revealing significantly higher expression in AML patients (Fig. 3B). Consistently, analysis of Gene Expression Profiling Interactive Analysis 2 (GEPIA2) data demonstrated that high MYC expression is associated with poorer prognosis in AML patients (Fig. 3C). Western blot analysis showed that the combination treatment more effectively suppressed c-MYC protein levels in MV4-11 cells than either single agent alone (Fig. 3D), even in the presence of the proteasome inhibitor MG132 (Fig. 3E). Consistent results were also observed in HL60 cells (Fig. S4C, D).

Surface plasmon resonance (SPR) assay experiments were performed to assess chidamide binding to recombinant c-MYC, using 10058-F4 as a positive control, which showed concentration-dependent binding (Fig. S4E). Using this validated assay, chidamide was shown to bind to c-MYC in a concentration-dependent manner. In a pure buffer system, the dissociation constant (Kd) was determined to be 7.953E-7 M (Fig. 3F). Notably, in the presence of 10 μM cytarabine, the Kd decreased to 1.704E-7 M (Fig. 3G), suggesting enhanced binding affinity of chidamide to c-MYC.

Molecular docking analysis showed that LYS945 of MYC forms a hydrogen bond with chidamide, while PRO938, VAL941, and ALA948 of MYC establish Pi-Alkyl interactions. Additionally, LEU210 of MAX forms a Pi-Sigma interaction (Fig. 3H, I).

In the docking of cytarabine with the chidamide-MYC-MAX complex, cytarabine interacts with ASP220 and ARG212 of MAX through hydrogen bonds, with ASP216 and LYS213 via carbon-hydrogen bonds, and with ASP220 through a Pi-anion interaction (Fig. S4F, G). To confirm the predicted binding sites, MYC protein with point mutations were generated. Compared to wild-type MYC, the mutant proteins showed significantly reduced binding affinity for chidamide, with a Kd value of 3.116E-6 M (Fig. 3J).

Molecular dynamics (MD) simulations were performed to evaluate whether the combination of chidamide and cytarabine binds more effectively to the MYC–MAX complex than either drug alone. After 25 ns of simulation, both protein–ligand complexes reached equilibrium, demonstrating the stability and reliability of the models. Notably, the cytarabine–chidamide–MYC–MAX complex displayed a lower RMSD than the chidamide–MYC–MAX complex, suggesting that the dual-drug combination confers greater binding stability to the MYC–MAX complex (Fig. 3K). Throughout the simulations, the solvent-accessible surface area (SASA) of the protein–ligand complexes gradually decreased, reflecting enhanced protein compactness. Moreover, the SASA of the chidamide–MYC–MAX complex fluctuated more than that of the cytarabine–chidamide–MYC–MAX complex, suggesting improved stability with the dual-drug combination (Fig. 3L). Similarly, the radius of gyration (Rg) steadily decreased during the simulations, indicating a more compact protein structure. The Rg of the chidamide–MYC–MAX complex showed greater fluctuation than that of the cytarabine–chidamide–MYC–MAX complex, consistent with the SASA results and further supporting the enhanced stability of the dual-drug combination (Fig. 3M). Additionally, the average number of hydrogen bonds was 0.206 in the chidamide–MYC–MAX complex and 1.41 in the cytarabine–chidamide–MYC–MAX complex, indicating stronger hydrogen bonding in the dual-drug system (Fig. 3N). The binding free energies for the chidamide–MYC–MAX and cytarabine–chidamide–MYC–MAX complexes were calculated to be −141.672 kJ/mol and −234.619 kJ/mol, respectively, with electrostatic and van der Waals interactions serving as the primary contributors (Table S4).

### The MYC signaling pathway drives the upregulation of RRP9 expression in AML

**Fig. 4:**
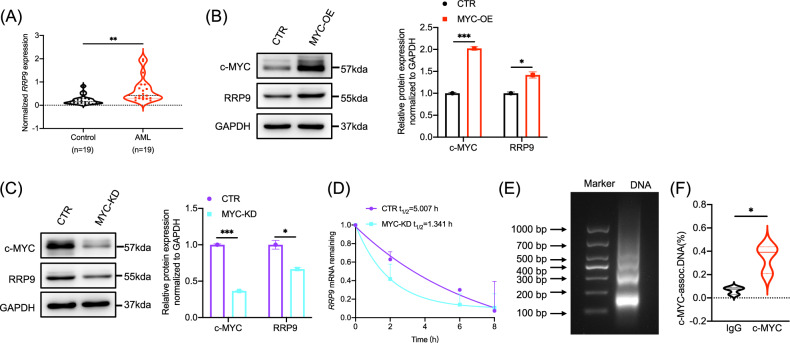
The MYC signaling pathway drives the upregulation of RRP9 expression in AML. **A** *RRP9* mRNA levels in PBMCs from AML patients and healthy individuals (*P* = 0.0019, unpaired t-test) (*n* = 19). **B** Western blot of c-MYC and RRP9 in MYC-overexpressing vs. control cells. Expression levels were significantly different for c-MYC (*P* = 0.0009) and RRP9 (*P* = 0.0165; unpaired t-test). **C** Western blot of c-MYC and RRP9 in MYC knockdown vs. control cells. Expression levels were significantly different for c-MYC (*P* = 0.0002) and RRP9 (*P* = 0.0180; unpaired t-test). **D** *RRP9* mRNA levels in MYC knockdown and control cells treated with actinomycin D over time (0 h, 2 h, 6 h, 8 h) detected by qPCR. **E** Agarose gel electrophoresis showing the size of purified DNA fragments. **F** ChIP-qPCR showing c-MYC enrichment at the RRP9 promoter relative to IgG (*P* = 0.0178, unpaired t-test). *P < 0.05, **P < 0.01, ***P < 0.001.

RNA-seq revealed that ribosome assembly factors were enriched in the MYC pathway (Fig. 3A). We further analyzed the expression of specific ribosome assembly factors—*RRP9*, *NOP16*, *NOP56*, and *DDX21*—in AML patients and healthy controls. Among them, only *RRP9* was significantly upregulated in AML patients (Fig. 4A), whereas *NOP16* and *NOP56* showed no significant changes (Fig. S5A, B), and *DDX21* was downregulated (Fig. S5C). To further explore MYC regulation of RRP9, we generated MYC-overexpressing and MYC-knockdown cell models. Western blot analysis showed that RRP9 protein levels increased with MYC overexpression (Fig. 4B) and decreased with MYC knockdown (Fig. 4C), consistent with c-MYC expression.

Using actinomycin D to assess *RRP9* mRNA stability, we found that the half-life of *RRP9* mRNA in the control group was 5.007 hours, whereas MYC knockdown shortened it to 1.341 hours. This result indicates that MYC knockdown accelerates *RRP9* mRNA degradation and diminishes its stability (Fig. 4D). To further investigate the relationship between MYC and RRP9, chromatin immunoprecipitation (ChIP) assay was performed. Purified DNA samples analyzed by 2% agarose gel electrophoresis displayed fragments ranging from 150 to 900 bp, confirming effective DNA digestion (Fig. 4E). Subsequent qPCR analysis of DNA samples (IgG, ChIP, INPUT) using primers targeting the RRP9 promoter region revealed significantly higher c-MYC enrichment at the RRP9 promoter site compared to the IgG control (Fig. 4F). These findings demonstrate that c-MYC directly binds to the RRP9 promoter sequence, thereby regulating RRP9 expression.

### Elevated RRP9 expression is associated with poor prognosis in AML

**Fig. 5:**
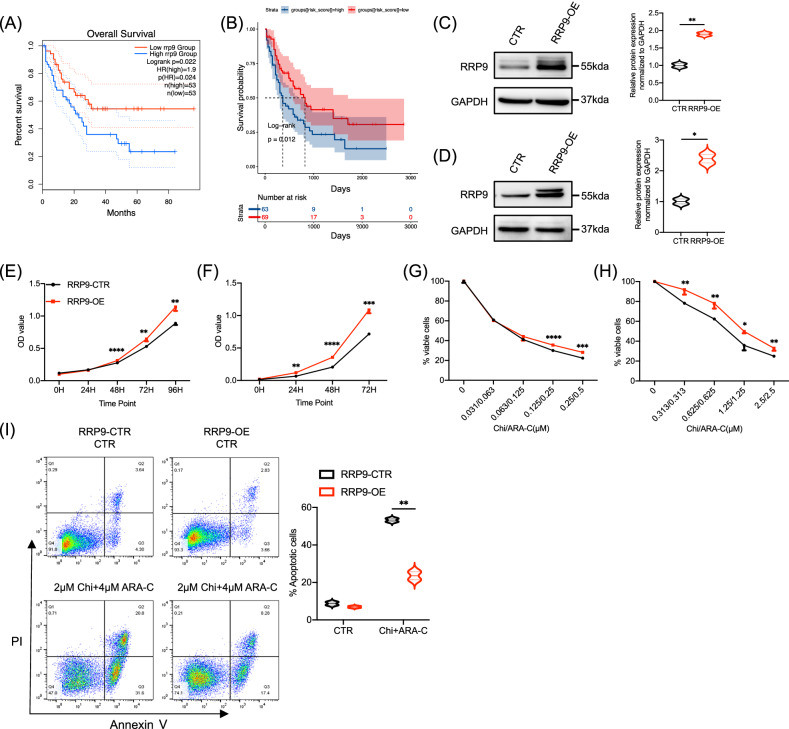
Elevated RRP9 expression is associated with poor prognosis in AML. **A** Data from the GEPIA2 website showing that high RRP9 expression in AML patients correlates with poor prognosis (*P* = 0.0240, Kaplan Meier analysis), median cutoff. (**B**), TCGA-LAML RNA-seq analysis revealing that high RRP9 expression is associated with poor prognosis in AML (*P* = 0.0120, Kaplan Meier analysis). **C**, **D** Western blot analysis revealed elevated RRP9 protein levels in the RRP9 overexpression group compared to the control in Kasumi-1 cells (above, *P* = 0.0052, unpaired t-test) and MV4-11 cells (below, *P* = 0.0132, unpaired t-test). **E**, **F** RRP9 overexpression significantly enhanced cell proliferation in Kasumi-1 cells (left; 48 h: *P* < 0.0001, 72 h: *P* = 0.0085, 96 h: *P* = 0.0029) and MV4-11 cells (right; 48 h: *P* = 0.0051, 72 h: *P* < 0.0001, 96 h: *P* = 0.0003), as determined by unpaired t-tests. **G**, **H** Overexpression of RRP9 reduced drug sensitivity in Kasumi-1 cells (left; *P* < 0.0001 and *P* = 0.0008 at two different concentrations) and MV4-11 cells (right; *P* = 0.0093, 0.0072, 0.0101 and 0.0062 at four different concentrations), as determined by unpaired t-tests. **I** Flow cytometry analysis showed that RRP9 overexpression enhanced drug resistance in Kasumi-1 cells (*P* = 0.0059, unpaired t-test). CTR (control), Chi (chidamide), ARA-C (cytarabine). *P < 0.05, **P < 0.01, ***P < 0.001, ****P < 0.0001.

Our analysis across multiple datasets consistently reveals that high RRP9 expression is a negative prognostic factor in AML. GEPIA2 data analysis demonstrated that high RRP9 expression correlates with poor prognosis in AML patients (Fig. 5A). Consistently, RNA-seq data from TCGA-LAML revealed that AML patients with elevated RRP9 expression had worse outcomes compared to those with lower expression (Fig. 5B). Similar findings were observed in additional datasets, including TARGET-AML RNA-seq (Fig. S5D) and microarray datasets GSE37642_GPL570 (Fig. S5E) and GSE12417_GPL96 (Fig. S5F), all linking high RRP9 expression to poor prognosis in AML.

We further explored the function of RRP9 by generating RRP9-overexpressing cell lines in Kasumi-1 and MV4-11 cells using lentiviral vectors to investigate its role in AML. Western blot analysis confirmed that RRP9 protein levels were significantly elevated in the overexpression group compared to the control in both Kasumi-1 (Fig. 5C) and MV4-11 cells (Fig. 5D). To assess the impact of RRP9 overexpression on AML cell proliferation, we performed CCK-8 assays and observed enhanced proliferation in RRP9-overexpressing cells relative to controls in both Kasumi-1 (Fig. 5E) and MV4-11 cells (Fig. 5F). Additionally, we evaluated the effect of RRP9 on drug sensitivity by treating the cells with chidamide and cytarabine. The CCK-8 assay revealed reduced toxicity of the combination treatment in RRP9-overexpressing cells, seen in both Kasumi-1 (Fig. 5G) and MV4-11 cells (Fig. 5H). Flow cytometry further showed that RRP9 overexpression resulted in less apoptosis following chidamide and cytarabine treatment (Fig. 5I).

### RRP9 regulates ribosome biogenesis in AML cells

**Fig. 6:**
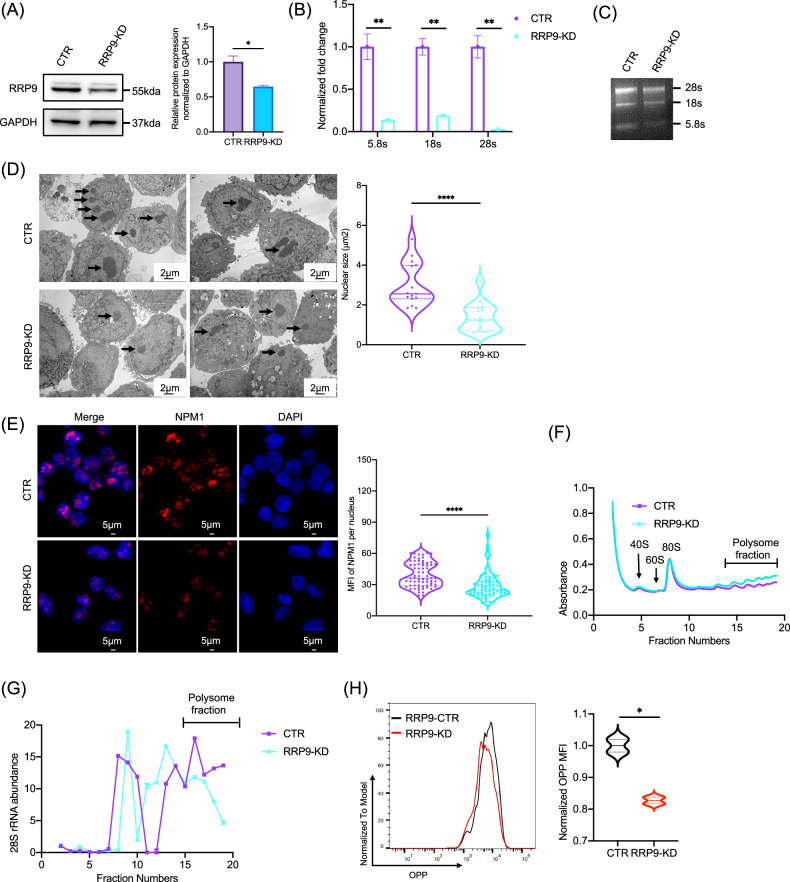
RRP9 regulates ribosome biogenesis in AML cells. **A** Western blot analysis showed reduced RRP9 protein levels in the RRP9 knockdown group compared to the control in Kasumi-1 cells, with statistical significance (*P* = 0.0290, unpaired t-test). **B** RRP9 knockdown significantly reduced mature rRNA abundance (5.8S: *P* = 0.0046, 18S: *P* = 0.0012, 28S: *P* = 0.0017, unpaired t-tests) in Kasumi-1 cells. **C** RNA gel electrophoresis further confirmed the reduction in mature rRNA levels following RRP9 knockdown. **D** Knockdown of RRP9 significantly reduced the nucleolar area (*P* < 0.0001, unpaired t-test) (*n* = 15). **E** Immunofluorescence analysis showed that the fluorescence intensity of NPM1 was significantly reduced after RRP9 knockdown (*P* < 0.0001, unpaired t-test) (*n* = 68). **F** Polysome fractionation assay revealed showing global mRNA translation levels between the control and RRP9 knockdown groups. **G** qPCR analysis of 28S rRNA translation levels across fractions. **H** RRP9 knockdown also decreased nascent protein synthesis (*P* = 0.0163, unpaired t-test). MFI Mean fluorescence intensity. *P < 0.05, **P < 0.01, ****P < 0.0001.

We next infected Kasumi-1 cells with lentivirus to generate RRP9 knockdown cell lines. Western blot analysis confirmed a reduction in RRP9 protein levels in the knockdown group (Fig. 6A). To evaluate the effect of RRP9 knockdown on mature rRNAs (5.8S, 18S, and 28S) abundance in AML cells, we performed qPCR, which revealed significant reductions in these rRNA species (Fig. 6B). RNA gel electrophoresis further confirmed the decreased abundance of mature rRNAs after RRP9 knockdown (Fig. 6C).

Additionally, transmission electron microscopy revealed a marked reduction in nucleolus size in the RRP9 knockdown cells (Fig. 6D). Consistent with this structural change, RRP9 knockdown significantly reduced the fluorescence intensity of NPM1 (Fig. 6E).

The proportion of ribosomes in polysomes is positively correlated with the translation initiation rate [31]. Analysis of polysome fractions showed no significant difference in overall mRNA translation levels between the control and RRP9 knockdown cell lines (Fig. 6F). However, qPCR analysis of specific mRNAs in each polysome fraction showed that 28S rRNA polysome absorbance was significantly reduced in the RRP9-KD group, indicating that RRP9 knockdown resulted in reduced 28S rRNA translation levels in Kasumi-1 cells (Fig. 6G). Additionally, nascent protein synthesis assays demonstrated that the fluorescence intensity of de novo protein synthesis was significantly lower in the RRP9-knockdown group than in the controls (Fig. 6H).

### Chidamide combined with cytarabine inhibits ribosome biogenesis in AML cells by downregulating RRP9

**Fig. 7:**
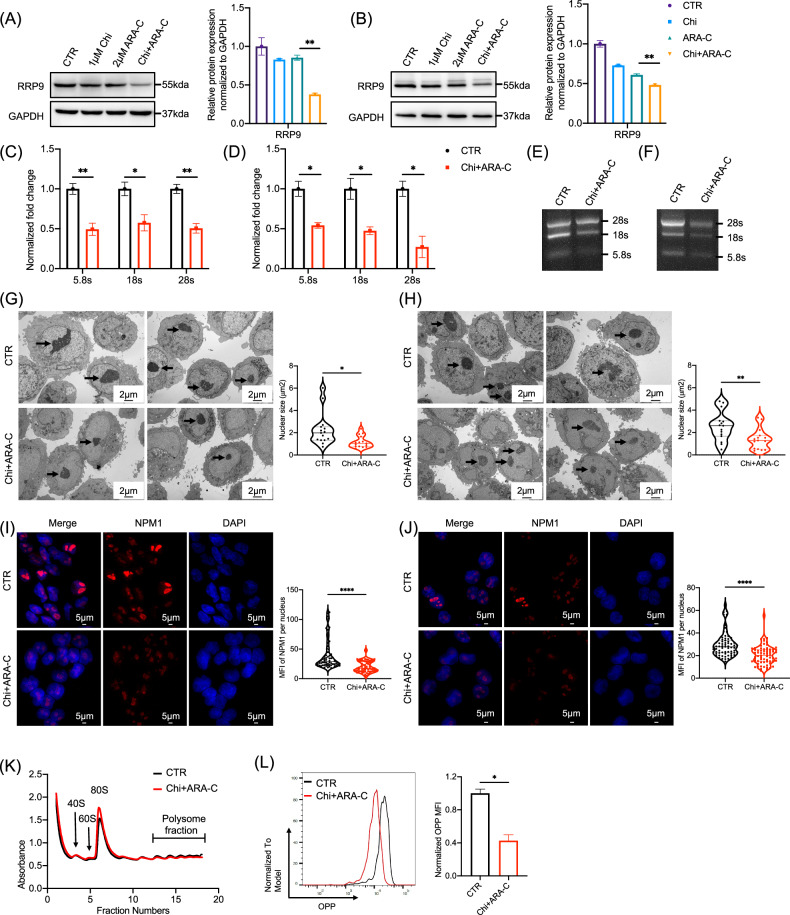
Chidamide combined with cytarabine inhibits ribosome biogenesis in AML cells by downregulating RRP9. **A**, **B** Effects of chidamide and cytarabine on RRP9 protein levels in MV4-11 (left, *P* = 0.0029) and Kasumi-1 cells (right, *P* = 0.0064), showing a significant difference between cytarabine monotherapy and the combination treatment (unpaired t-test). **C**, **D** qPCR analysis showed that treatment with chidamide and cytarabine reduced mature rRNA abundance (5.8S, 18S, and 28S) in MV4-11 cells (left; 5.8S: *P* = 0.0078, 18S: *P* = 0.0332, 28S: *P* = 0.0041) and Kasumi-1 cells (right; 5.8S: *P* = 0.0105, 18S: *P* = 0.0202, 28S: *P* = 0.0115), as determined by unpaired t-tests. **E, F** RNA gel electrophoresis confirmed reduced mature rRNA levels in MV4-11 cells and Kasumi-1 cells after chidamide and cytarabine treatment. **G**, **H** Electron microscopy revealed that treatment with chidamide and cytarabine altered nucleolar morphology in MV4-11 cells (left, *P* = 0.0112, unpaired t-test) and Kasumi-1 cells (right, *P* = 0.0093, unpaired t-test) (*n* = 15). **I, J** Immunofluorescence analysis showed that the fluorescence intensity of NPM1 in MV4-11 cells (left) and Kasumi-1 cells (right) was significantly decreased after treatment with chidamide and cytarabine (*P* < 0.0001, unpaired t-test) (*n* = 68). **K** Polysome fractionation assay showed an increase in the 80S peak in MV4-11 cells treated with chidamide and cytarabine. **L** The OPP kit and flow cytometry analysis showed a decrease in nascent protein synthesis in Kasumi-1 cells after combination treatment (*P* = 0.0221, unpaired t-test). Drug concentrations were 1 µM chidamide and 2 µM cytarabine, administered for 24 h CTR (control), Chi+ARA-C (chidamide + cytarabine). MFI: Mean fluorescence intensity. *P < 0.05, **P < 0.01, ****P < 0.0001.

Based on our findings that chidamide combined with cytarabine inhibits the MYC-RRP9 pathway (Figs. 3 and 4) and that reduced RRP9 impairs ribosome biogenesis in AML (Fig. 6), we further examined the effects of this combination on ribosome biogenesis. Western blot showed that the combined treatment more effectively reduced RRP9 protein levels than either chidamide or cytarabine alone in both MV4-11 (Fig. 7A) and Kasumi-1 cells (Fig. 7B). RRP9 expression in HL60 cells was also decreased (Fig. S6A). qPCR analysis showed that the abundance of mature rRNAs was reduced after combination treatment in both cell lines (Fig. 7C, D), which was confirmed by RNA gel electrophoresis (Fig. 7E, F). Electron microscopy showed that the nucleolar area was reduced in MV4-11 and Kasumi-1 AML cells following dual-drug treatment (Fig. 7G, H). Immunofluorescence analysis further revealed decreased NPM1 fluorescence intensity in these two cell lines (Fig. 7I, J), with similar results observed in HL60 cells (Fig. S6B). In double-drug-treated MV4-11 cells, an increase in 80S ribosome absorbance compared to the control group was observed (Fig. 7K), indicating translation arrest. Consistently, nascent protein synthesis assays showed a marked decrease in fluorescence intensity of nascent proteins in Kasumi-1 cells, further supporting this conclusion (Fig. 7L).

## Discussion

With advances in sequencing technology, our understanding of the molecular mechanisms underlying AML has grown significantly, uncovering genetic and clonal heterogeneity, and laying the groundwork for precision medicine. The introduction of targeted inhibitors for FLT3, IDH1/IDH2, and BCL-2 exemplifies the progress of precision therapies. While these drugs demonstrate moderate anti-leukemia activity when used alone, their clinical efficacy is markedly improved when combined with agents targeting epigenetic or other oncogenic pathways, or traditional cytotoxic treatments [32]. This underscores the potential of precision medicine to enhance clinical outcomes for AML patients. Identifying additional therapeutic targets further expands the promise of personalized treatment for AML.

In this study, we show that the combination of chidamide and cytarabine exerts a synergistic therapeutic effect against AML, both in vitro and in vivo. This promising approach could represent a significant advancement in the treatment of this aggressive cancer. This finding aligns with previous studies [33–35] and further highlights the potential of this combination therapy. Our study demonstrates that chidamide and cytarabine not only jointly suppress the MYC pathway, but also synergistically bind directly to MYC. Zhang et al. demonstrated that MYC is crucial to AML pathogenesis by sustaining the undifferentiated state and self-renewal capacity of leukemic stem cells [36]. Although no MYC-targeting compounds have yet entered clinical trials, APTO-253, which partially reduces MYC expression, is currently undergoing phase I trials for relapsed or refractory AML or myelodysplastic syndromes (NCT02267863) [37]. These findings suggest an encouraging clinical outlook for MYC inhibition using chidamide in combination with cytarabine for AML.

Our studies reveal that MYC serves as an upstream regulator of RRP9. Elevated RRP9 expression is associated with poor prognosis in AML, while decreased RRP9 levels exert therapeutic effects on AML by impairing ribosome biogenesis. These findings underscore the critical role of RRP9 in ribosome biogenesis and highlight its potential as a target for cancer therapy. Cancer cells often upregulate ribosome biogenesis to meet increased protein synthesis demands [14], and targeting this process has emerged as a promising therapeutic strategy [38, 39]. In AML, enhanced translation initiation has been shown to promote tumor growth [40], while inhibitors of protein translation effectively target leukemic cells [24]. Moreover, reduced protein synthesis and ribosome instability can trigger apoptosis in leukemic cells [41]. Together, these insights position RRP9 as a key player in ribosome biogenesis and a promising target for AML therapy.

Finally, we found that the combination of chidamide and cytarabine synergistically suppresses ribosome biogenesis in AML cells by downregulating RRP9. This finding underscores the significance of targeting key oncoproteins and critical signaling pathways, paving the way for optimizing AML treatment strategies through combination therapies. Further research is required to fully understand the molecular interactions and refine the clinical application of this promising approach. However, this study has some limitations. We mainly focused on the MYC-RRP9-ribosome biogenesis axis. Whether it relates to known mechanisms of chidamide and cytarabine or represents a novel pathway needs further study.

In conclusion, this study offers a more comprehensive understanding of the synergistic therapeutic effects and underlying mechanisms of chidamide combined with cytarabine in AML, both in vitro and in vivo. The combination of chidamide and cytarabine induces AML cell death by inhibiting ribosome biogenesis through downregulation of the MYC-RRP9 pathway.

## Materials and Methods

### Cell lines and reagents

The AML cell lines MV4-11, Kasumi-1, and HL60 were obtained from ATCC (Manassas, VA, USA). Their authenticity was confirmed by STR profiling, and all experiments used mycoplasma-free cultures.

Chidamide was provided by Chipscreen Biosciences (Shenzhen, China). Cytarabine, puromycin 2HCl, and 10058-F4 were purchased from Selleck Chemicals (Houston, USA), and MG132 from Meilun Biotech (Dalian, China).

### SPR assay

For SPR measurements, c-MYC protein (RayBiotech, USA) was dissolved in double-distilled water, diluted to 25 μg/ml with sodium acetate buffer (pH4.5), and immobilized on a CM5 chip ( ~ 4000 RU) with a blank channel as control. Chidamide was applied at concentrations from 13.7 nM to 10 μM over immobilized c-MYC at 30 μl/min, and binding was recorded. Kinetic analysis and Kd determination were performed using a Biacore X100 with the Steady-State Affinity model. For synergy experiments, cytarabine was prepared in buffer at a concentration of 10 μM. To validate the docking sites, a recombinant MYC protein carrying the predicted substitutions (P406A, V409A, K413A, A416G) was obtained from MedChemExpress (HY-P7S1056, USA).

### Other Methods

The detailed protocols for MTT assays, flow cytometry, western blot (all full-length and uncropped blots are shown in Fig. S7), mononuclear cell isolation, CCK-8 assays, animal studies, RNA-seq, qPCR, gene expression-prognosis link in AML, molecular docking, MD simulations, lentivirus construction and infection, mRNA stability assay, ChIP assay, RNA gel electrophoresis, electron microscopy observation of nucleoli, immunofluorescence, polysome fractionation assay [42], and nascent protein detection assays can be found in the Supplemental Materials and Methods. RNA-seq coverage and quality statistics are shown in Table S5. qPCR primer sequences for 5.8S, 18S, 28S rRNA [43], RRP9 promoter, NOP16, NOP56, and DDX21 are provided in Table S6.

### Statistical analysis

Data are shown as mean ± standard deviation. Western blot and immunofluorescence were quantified with ImageJ, and statistical analysis was performed using GraphPad Prism 8.0. t-tests were used, with *P*< 0.05 considered significant (*).

## Supplementary information

Fig.S7_wb

Supplemental Material

## Data Availability

The Raw and normalized RNA-seq data generated in this study are publicly available in Gene Expression Omnibus under accession number GSE231914 (https://www.ncbi.nlm.nih.gov/geo/query/acc.cgi?acc=GSE231914). All other data are available in the main text or supplementary materials.
