# Supplementary material for: Chidamide and cytarabine synergistically treat acute myeloid leukemia: inhibiting ribosome biogenesis via the MYC-RRP9 pathway
Source: Cell Death Dis. 2025 Aug 9;16(1):601. doi: 10.1038/s41419-025-07928-y (PMC12334614; doi:10.1038/s41419-025-07928-y)
Supplement: Supplementary file 1 — Fig.S7_wb [file 41419_2025_7928_MOESM1_ESM.pdf]

Figure 1

(G)

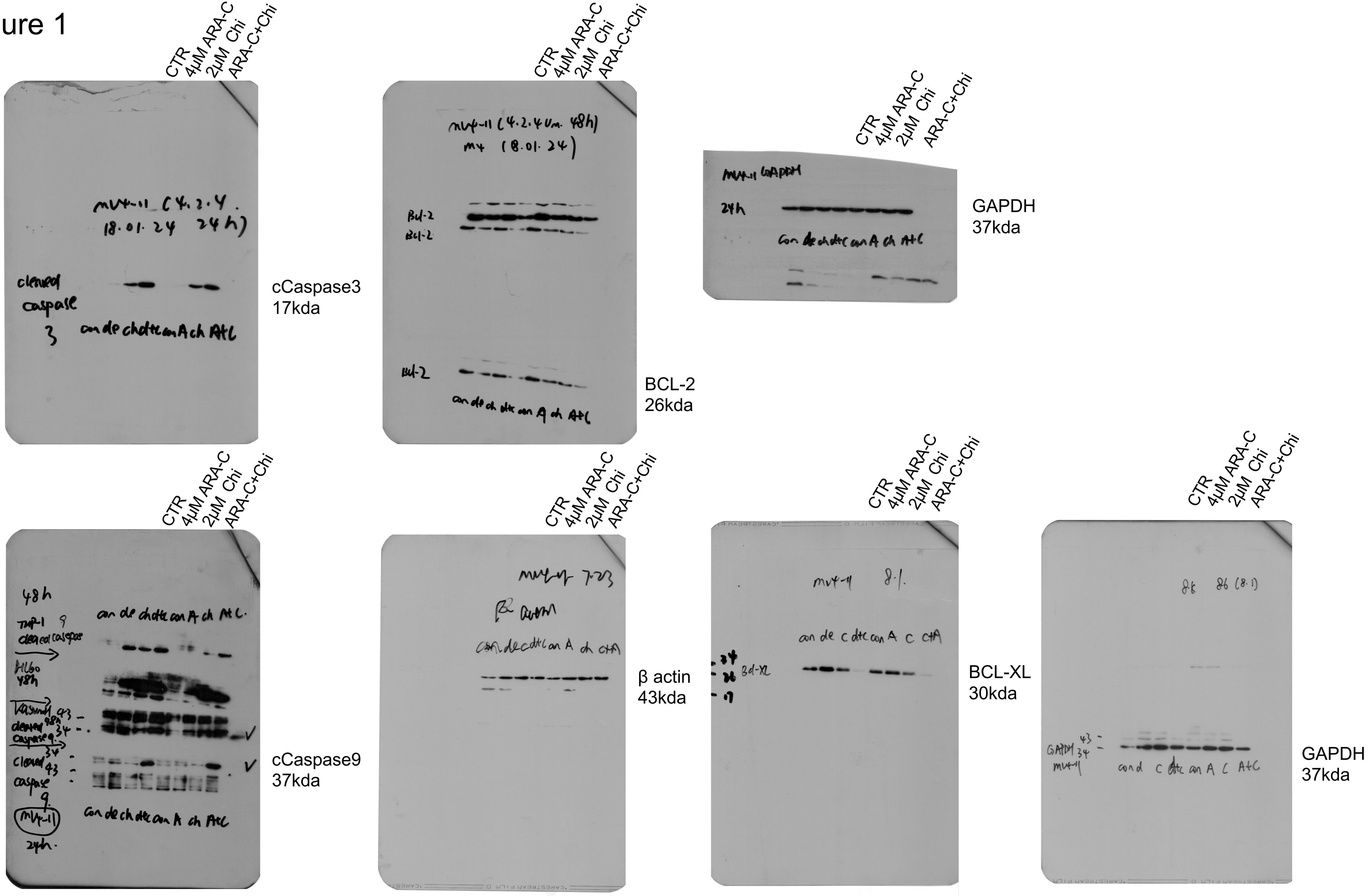
CTR
4μM ARA-C
2μM Chi
ARA-C+Chi
cCaspase3
17kda
BCL-2
26kda
GAPDH
37kda
cCaspase9
37kda
β actin
43kda
BCL-XL
30kda
GAPDH
37kda

Figure 1

(H)

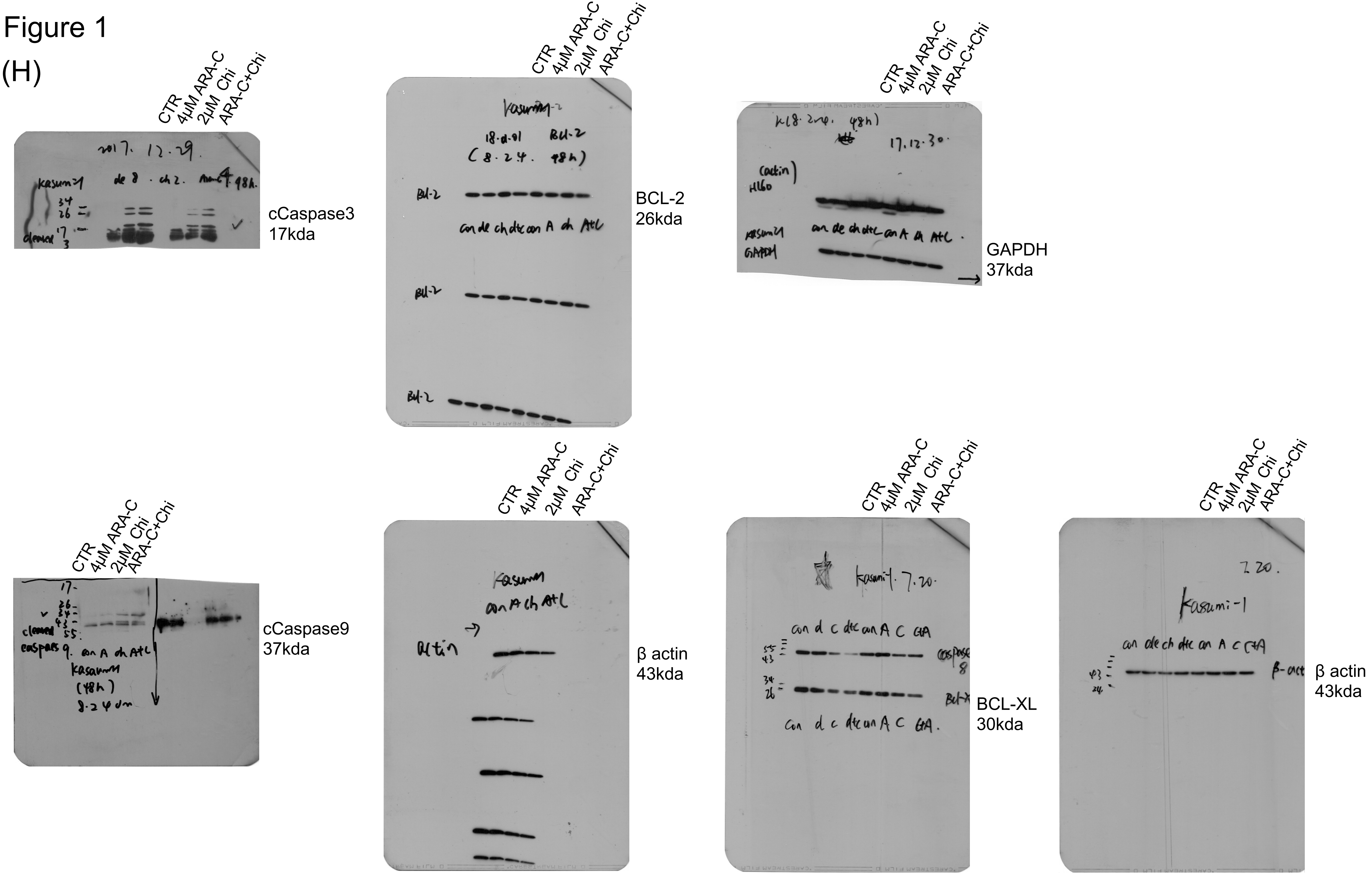
CTR
4μM ARA-C
2μM Chi
ARA-C+Chi
cCaspase3
17kda
BCL-2
26kda
GAPDH
37kda
cCaspase9
37kda
β actin
43kda
BCL-XL
30kda
β actin
43kda

Figure 3

(D)

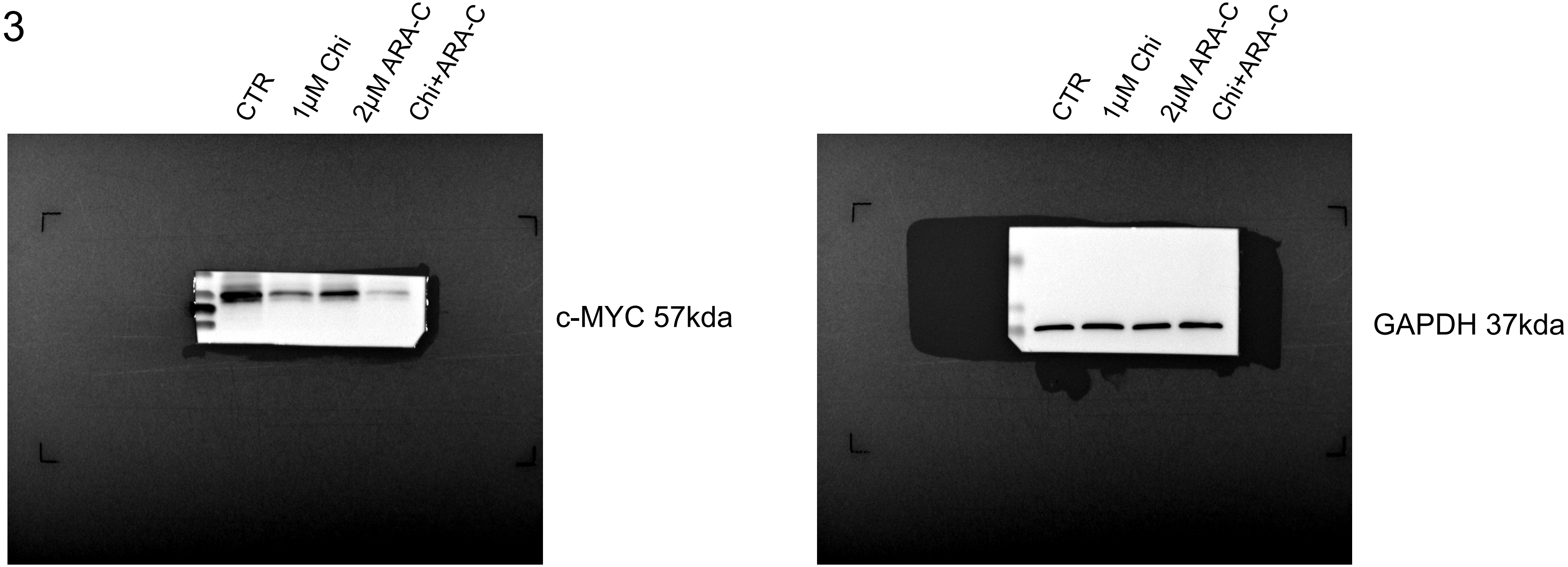
CTR
1μM Chi
2μM ARA-C
Chi+ARA-C
c-MYC 57kda
CTR
1μM Chi
2μM ARA-C
Chi+ARA-C
GAPDH 37kda

(E)

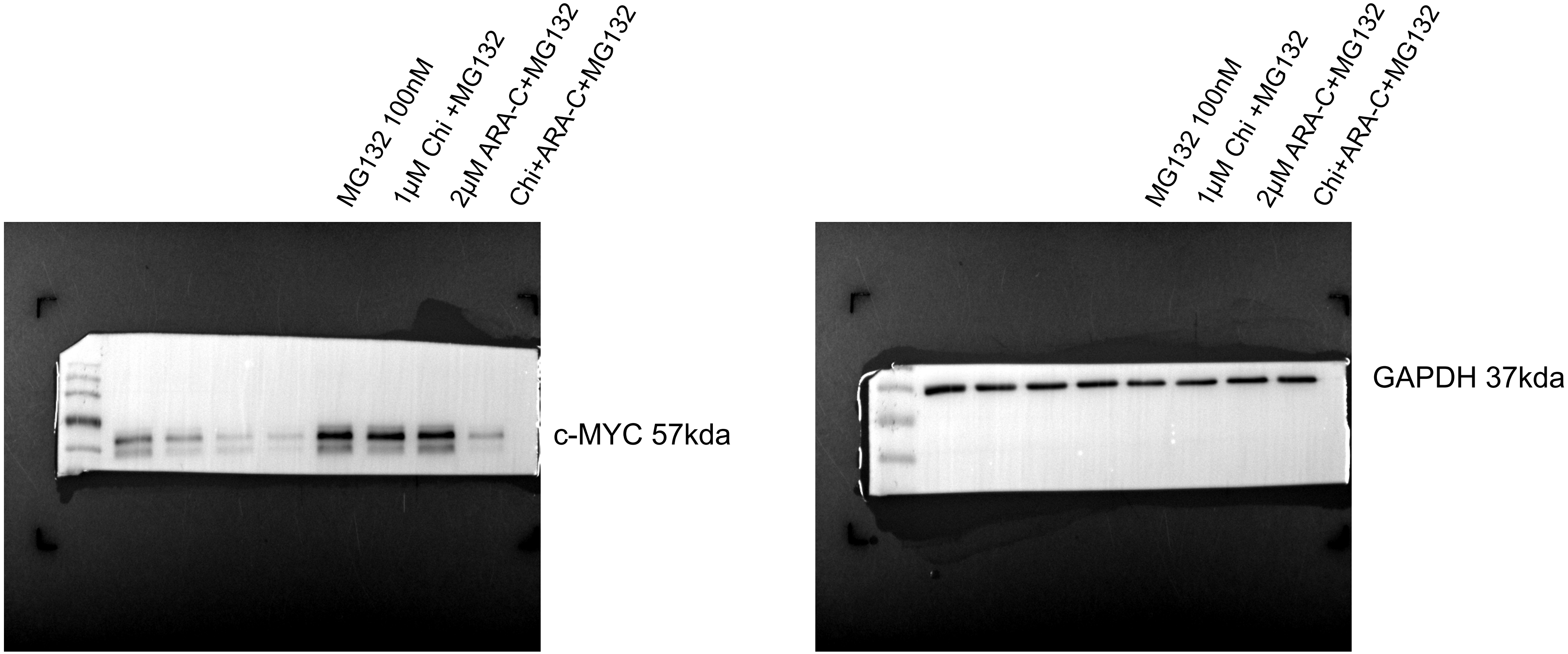
MG132 100nM
1μM Chi +MG132
2μM ARA-C+MG132
Chi+ARA-C+MG132
c-MYC 57kda
MG132 100nM
1μM Chi +MG132
2μM ARA-C+MG132
Chi+ARA-C+MG132
GAPDH 37kda

Figure 4

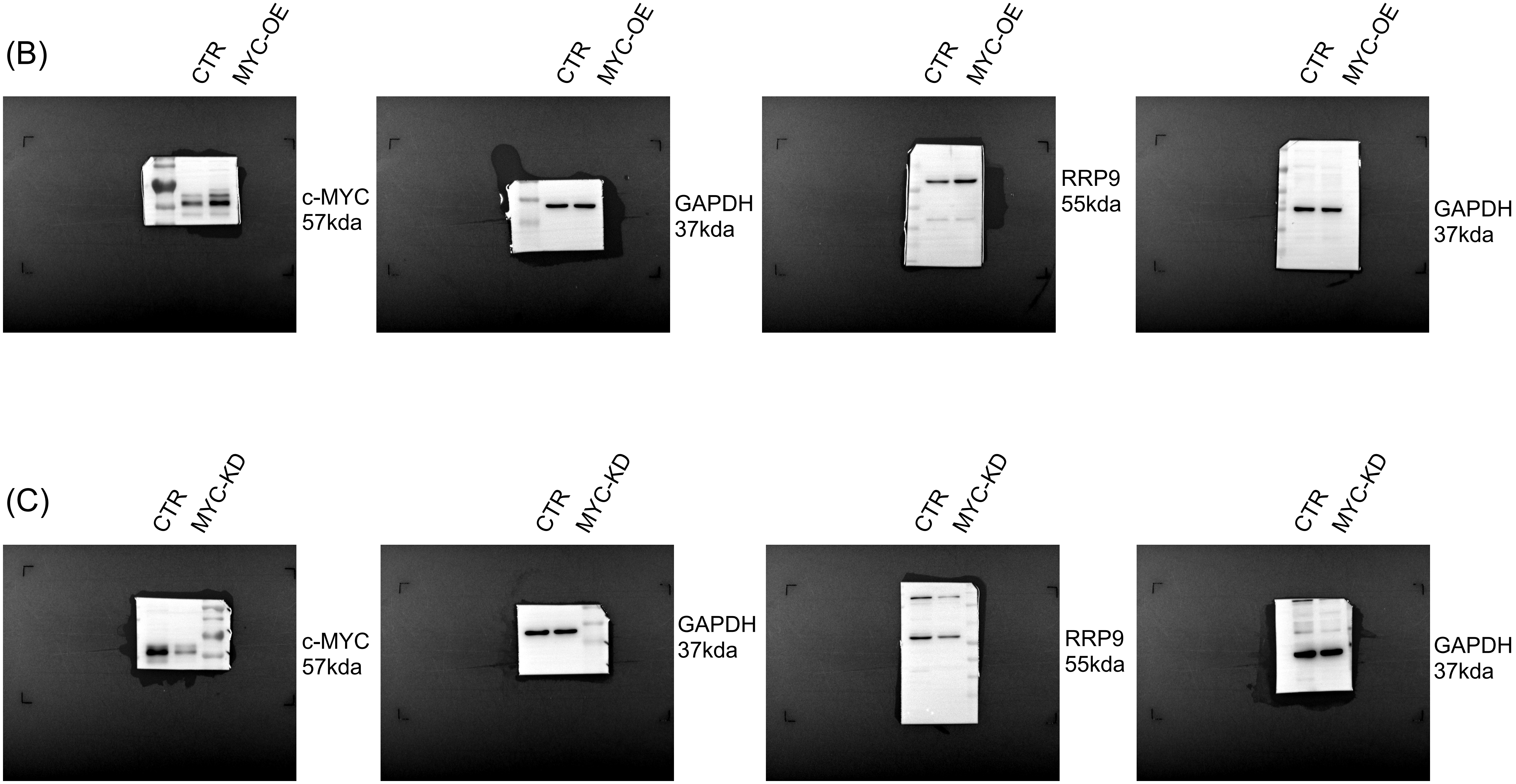

(B)
CTR
MYC-OE
c-MYC
57kda
GAPDH
37kda
RRP9
55kda
GAPDH
37kda
(C)
CTR
MYC-KD
c-MYC
57kda
GAPDH
37kda
RRP9
55kda
GAPDH
37kda

Figure 5

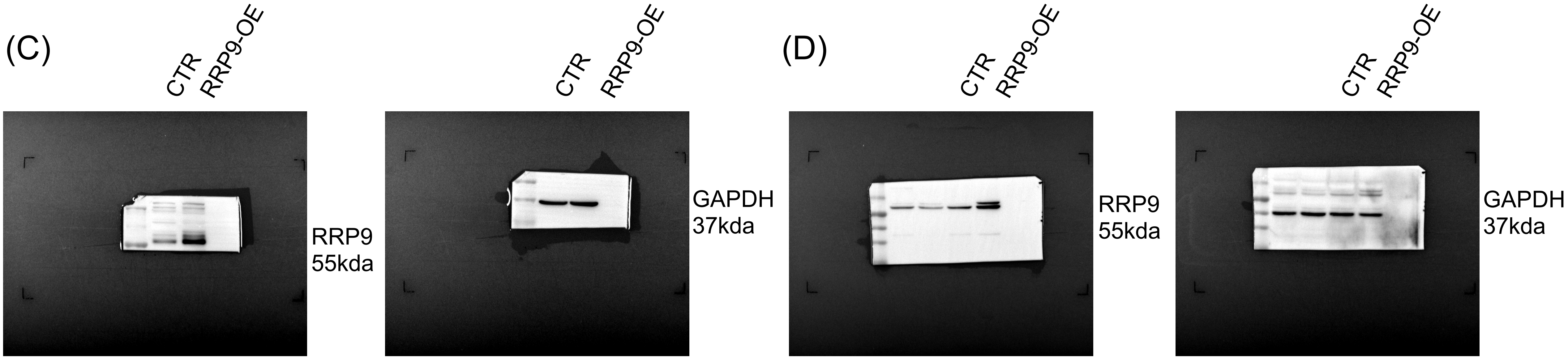

Figure 6

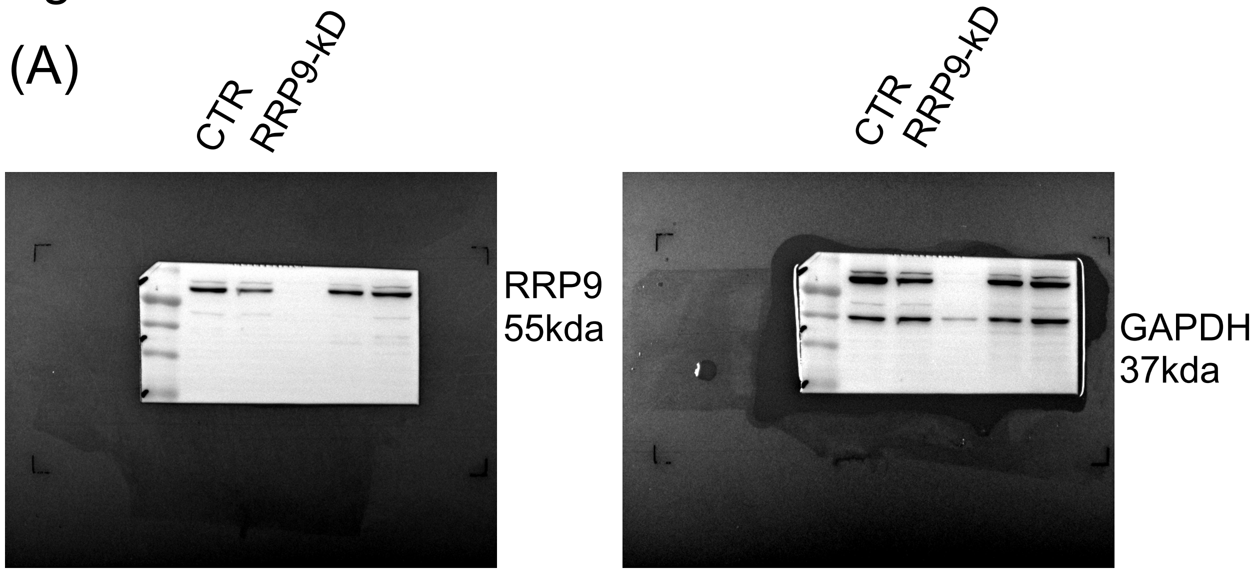

Figure 7

(A)

(B)

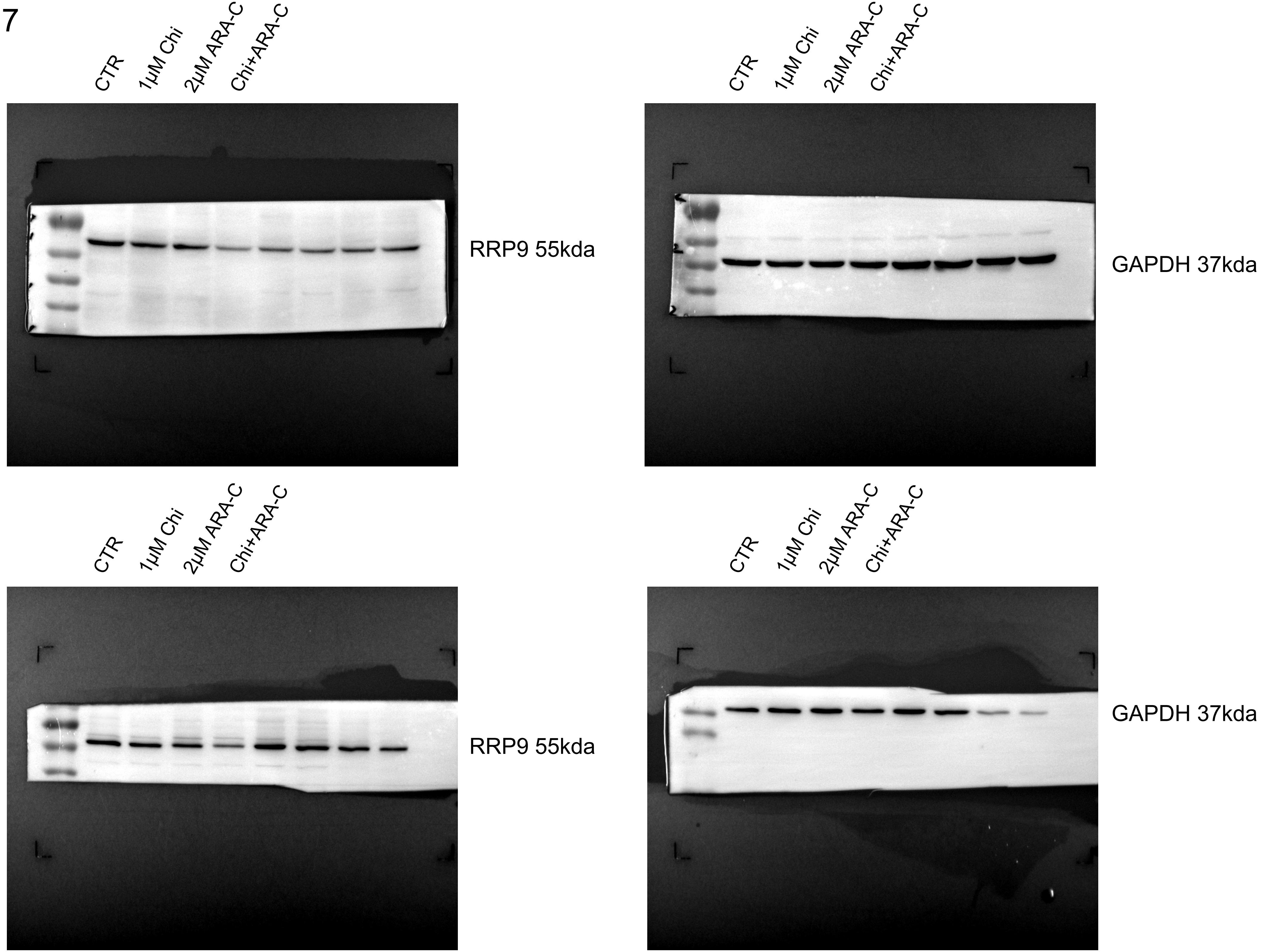

Figure S2

(D)

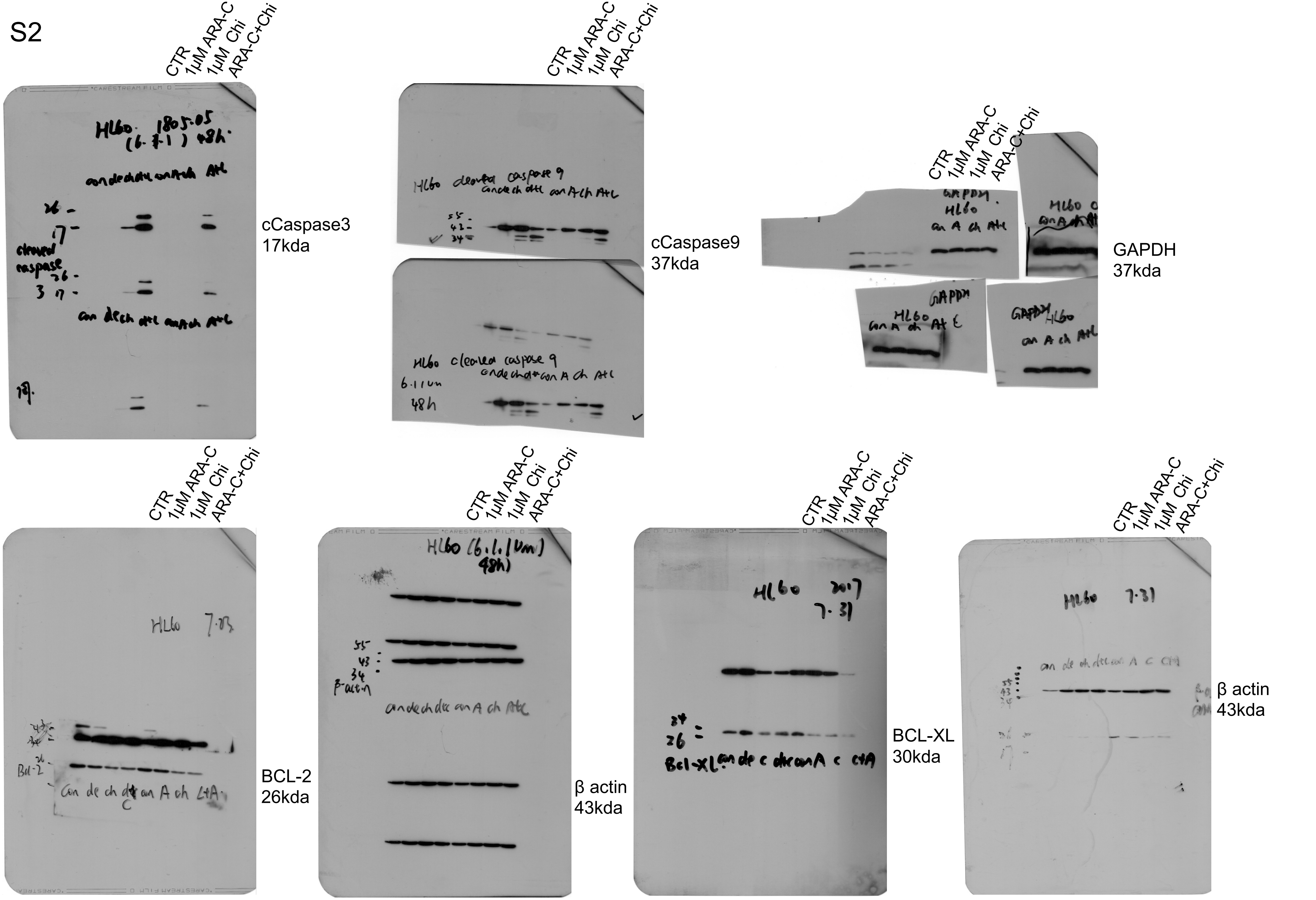
CTR
1μM ARA-C
1μM Chi
ARA-C+Chi
cCaspase3
17kda
cCaspase9
37kda
GAPDH
37kda
BCL-2
26kda
β actin
43kda
BCL-XL
30kda
β actin
43kda

Figure S3

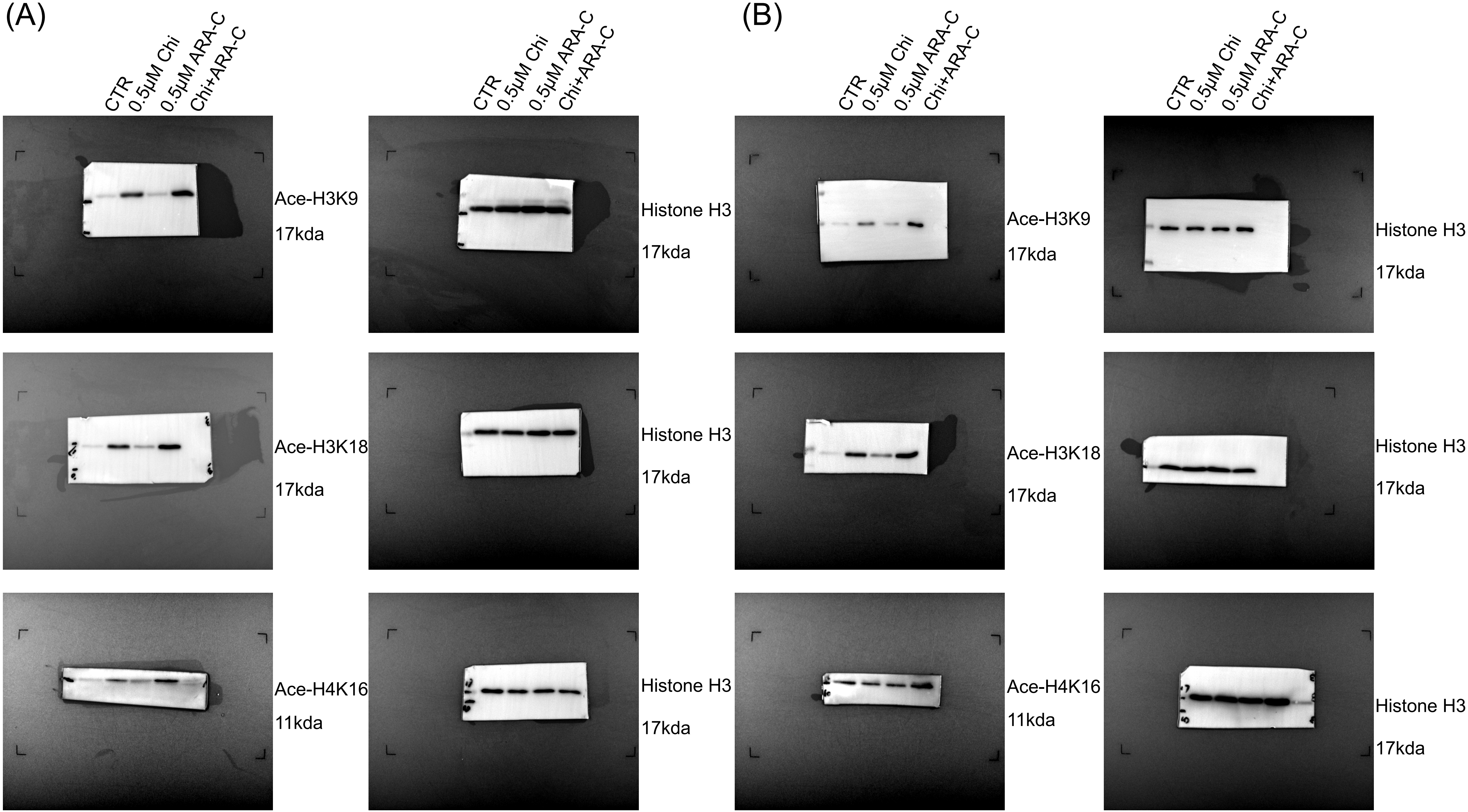
(A)
CTR
0.5μM Chi
0.5μM ARA-C
Chi+ARA-C
Ace-H3K9
17kda
Histone H3
17kda
Ace-H3K18
17kda
Histone H3
17kda
Ace-H4K16
11kda
Histone H3
17kda
(B)
CTR
0.5μM Chi
0.5μM ARA-C
Chi+ARA-C
Ace-H3K9
17kda
Histone H3
17kda
Ace-H3K18
17kda
Histone H3
17kda
Ace-H4K16
11kda
Histone H3
17kda

Figure S4

(C)

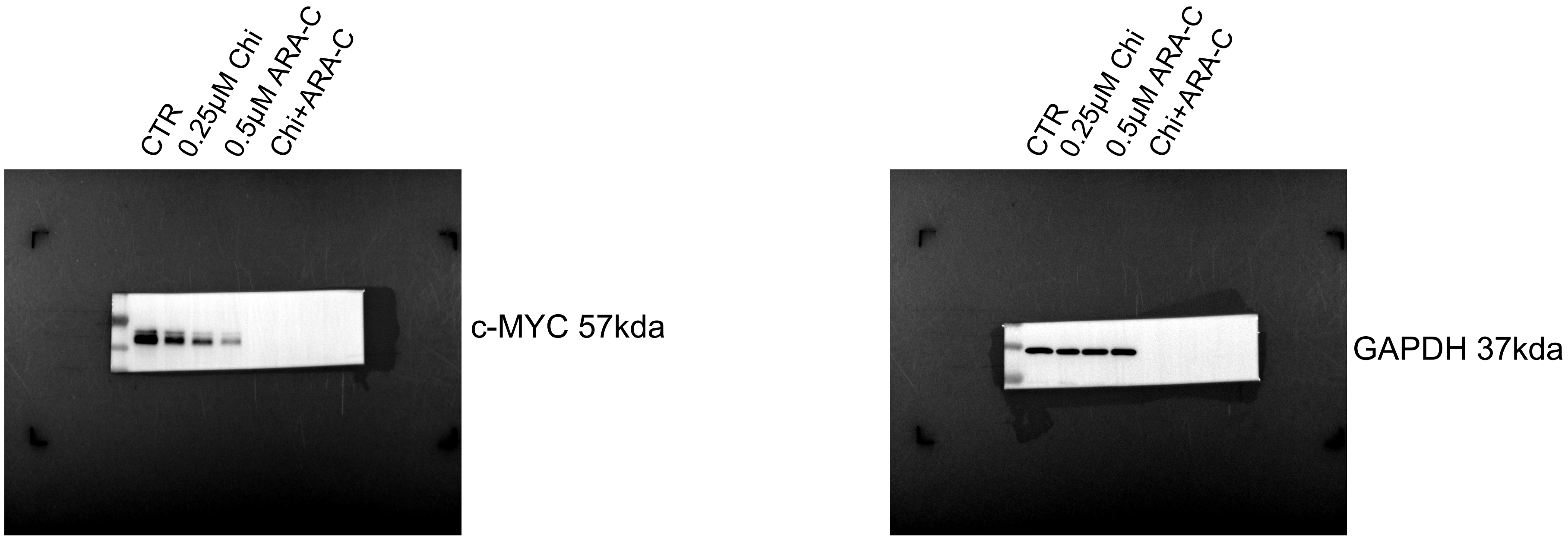
CTR
0.25μM Chi
0.5μM ARA-C
Chi+ARA-C
c-MYC 57kda
CTR
0.25μM Chi
0.5μM ARA-C
Chi+ARA-C
GAPDH 37kda

(D)

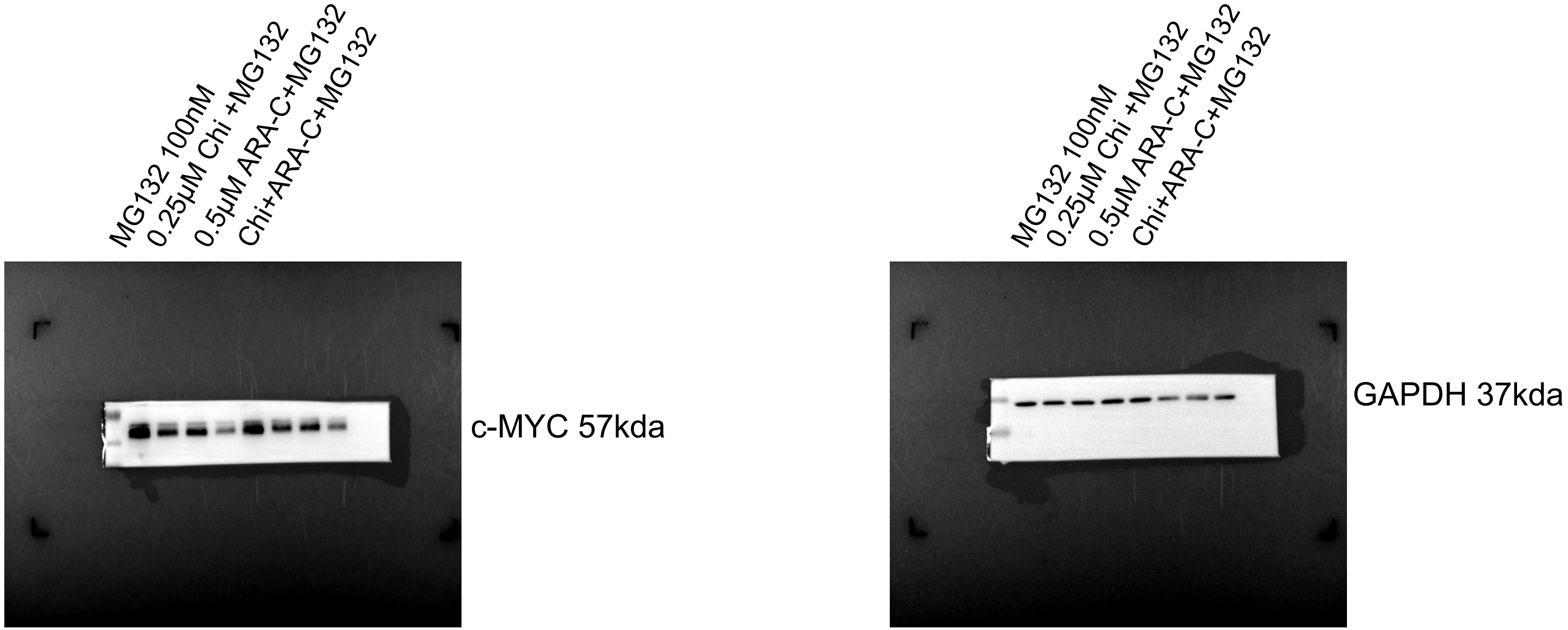
MG132 100nM
0.25μM Chi +MG132
0.5μM ARA-C+MG132
Chi+ARA-C+MG132
c-MYC 57kda
MG132 100nM
0.25μM Chi +MG132
0.5μM ARA-C+MG132
Chi+ARA-C+MG132
GAPDH 37kda

Figure S6

(A)

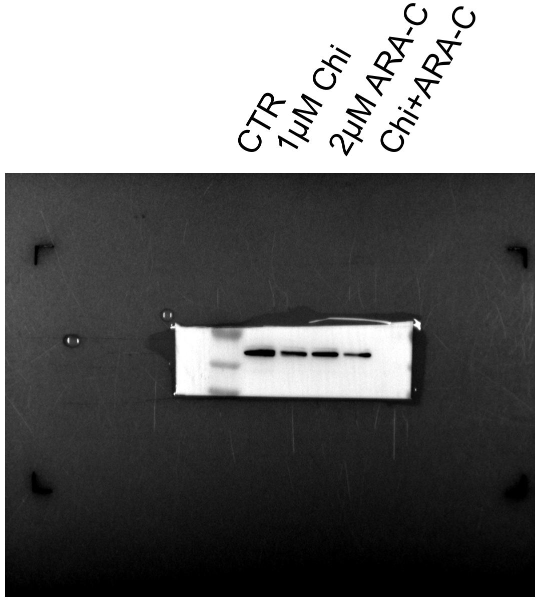

RRP9 55kda

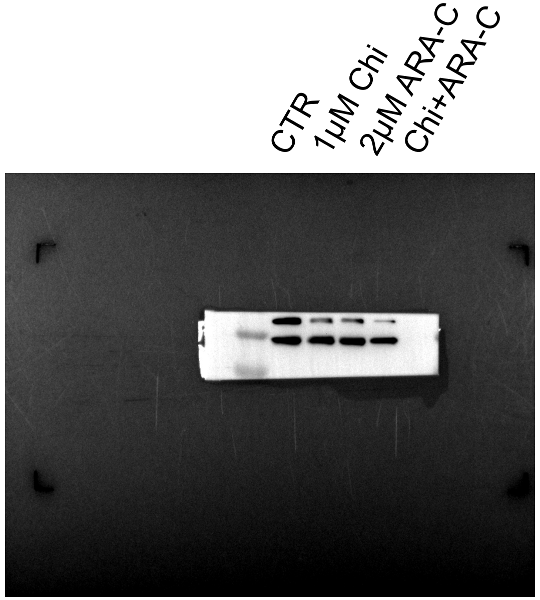

GAPDH 37kda
