# Supplementary material for: Chidamide and cytarabine synergistically treat acute myeloid leukemia: inhibiting ribosome biogenesis via the MYC-RRP9 pathway
Source: Cell Death Dis. 2025 Aug 9;16(1):601. doi: 10.1038/s41419-025-07928-y (PMC12334614; doi:10.1038/s41419-025-07928-y)
Supplement: Supplementary file 2 — Supplemental Material [file 41419_2025_7928_MOESM2_ESM.pdf]

Supplemental Material

Title

Chidamide and cytarabine synergistically treat acute myeloid leukemia: inhibiting ribosome biogenesis via the MYC-RRP9 pathway

Authors

Qing Li et al.

Corresponding author:

Yu Wu M.D., Ph.D. E-mail: wu_yu@scu.edu.cn

Materials and Methods

MTT assay

AML cells were cultured in 100 μL of RPMI-1640 or IMDM supplemented with 10% fetal bovine serum and treated with specific drug concentrations for 24, 48, and 72 hours in triplicate. After incubation, 20 μL of MTT reagent (5 mg/mL, Biosharp Life Sciences, China) was added and cells were incubated at 37°C for an additional 4 hours. Then, 100 μL of formazan dissolve solution was added and the mixture was incubated at 37 °C overnight. The absorbance was measured at 570 nm, and the half inhibitory concentration (IC50) was calculated using IBM SPSS Statistics, and the combination index (CI) was calculated using CompuSyn software.

Flow cytometry

Cells were washed twice with 1 mL of ice-cold PBS and resuspended in 200 μL of binding buffer. Next, 2 μL of Annexin V Alexa Fluor 647 and 1 μL of PI (4A Biotech, China) were added and incubated for 15 minutes at room temperature, shielded from light. Apoptosis was assessed using a Navios flow cytometer (Beckman Coulter, USA).

Western blot

Proteins were extracted using RIPA buffer (Beyotime Biotechnology, China) supplemented with protease inhibitors (Roche, Germany), and quantified via the BCA assay (Beyotime Biotechnology, China). After denaturation, samples were separated by SDS-PAGE and transferred to PVDF membranes (Millipore, USA). Membranes were blocked with 5% skim milk for 1 hour at room temperature and incubated overnight at 4°C with primary antibodies against cleaved caspase-3 (9664S), cleaved caspase-9 (20750S), BCL-2 (3498T), BCL-XL (2764T), Ace-H3K9 (9649S), Ace-H3K18 (13998S), Ace-H4K16 (13534S), Histone H3 (9715S), c-MYC (5605P, all from Cell Signaling Technology, USA), RRP9 (A303-970A, Bethyl Laboratories, USA), and GAPDH (TA309157, ZSGB-Bio, China). After washing with TBST, membranes were incubated with secondary antibodies (ZB-2305 or ZB-2306, ZSGB-Bio, China), and protein signals were visualized using a chemiluminescence detection kit (Millipore, USA).

Mononuclear cell isolation

The extraction of primary AML cells was approved by the Ethics Committee of West China Hospital, Sichuan University (approval number: 2019 (114)). Peripheral blood (2-3 mL) from AML patients or healthy donors was processed with Ficoll lymphocyte separation solution (TBD Science, China) to isolate peripheral blood mononuclear cells (PBMCs). The PBMCs were mixed with five volumes of PBS and centrifuged at 800 rpm for 5 minutes. After erythrocyte lysis using erythrocyte lysis buffer (Leagene, China), the PBMCs were resuspended in PBS and thoroughly mixed.

CCK-8 assay

Treated cells were incubated with 10 μL of CCK-8 solution (4A Biotech, China) at 37°C for 3 hours in a 5% CO2 incubator. Absorbance was measured at 490 nm using a SpectraMax 190 microplate reader (Molecular Devices, USA).

Animal studies

The animal experiments were approved by the Ethics Committee of West China Hospital, Sichuan University (approval number: 2020232A). Severely immunodeficient female B-NDG mice (6–8 weeks old) from Biocytogen Biotechnology Co., Ltd. (Beijing, China) were housed at the SPF-level Laboratory Animal Center, Sichuan University. The sample size, determined through prior studies and power analysis, included 5 mice per group. The mice were randomly assigned to experimental groups, with 5 mice per group, to ensure unbiased distribution. However, blinding was not applied in the assignment of treatment groups. The mice were intravenously injected with MV4-11-luciferase-GFP cells, generated by infecting MV4-11 cells with a lentivirus carrying luciferase and GFP. Tumor progression was monitored using an IVIS Spectrum In-Vivo Imaging System (PerkinElmer, USA), and data analysis was conducted with Living Image software (PerkinElmer, USA). To assess the impact of drug treatments on tumor burden, mice received intragastric doses of 5 mg/kg chidamide twice a week, and 20 mg/kg cytarabine was administered intraperitoneally daily for 5 days.

RNA-seq

MV4-11 cells were treated with 1 µM chidamide, 2 µM cytarabine, or a combination of both for 24 hours. The treated cells were collected, lysed using Trizol, and processed for RNA sequencing by Novogene (Beijing, China). A total of 1 µg RNA per sample was used as input for library preparation. cDNA fragments, preferentially between 370–420 bp in length, were selected and purified using the AMPure XP system (Beckman Coulter, Beverly, USA). PCR amplification was performed with Phusion High-Fidelity DNA polymerase, Universal PCR primers, and Index (X) Primer. PCR products were then purified (AMPure XP system), and library quality was assessed using the Agilent Bioanalyzer 2100 system. Index-coded samples were clustered on a cBot Cluster Generation System using the TruSeq PE Cluster Kit v3-cBot-HS (Illumina) according to the manufacturer's guidelines. Following cluster generation, the library was sequenced on an Illumina Novaseq platform, generating 150 bp paired-end reads. The reference genome index was built using Hisat2 v2.0.5 and paired-end clean reads were aligned to the reference genome using Hisat2 v2.0.5. Sequencing coverage and quality statistics are provided in Table S5. Gene set enrichment analysis was performed on gene_fpkm data from the sequencing results.

Quantitative real-time polymerase chain reaction (qPCR)

We used the CFX96 Real-Time PCR Detection System (Bio-Rad, USA) for reverse-transcription qPCR with 2X SYBR Green Fast qPCR Mix (Biomarker Technologies, China). Relative mRNA expression levels were calculated using the 2-ΔΔCt method. Primers were synthesized by Tsingke Biotechnology Co., Ltd. (Beijing, China) and are listed in Table S6.

Gene expression-prognosis link in AML

In this study, we explored the survival significance of MYC and RRP9 in AML by querying the GEPIA2 website (http://gepia2.cancer-pku.cn/#survival) and analyzing data from 10 publicly available human bulk AML datasets. RNA-seq data from The Cancer Genome Atlas adult de novo AML (TCGA-LAML) and TARGET-AML were downloaded from UCSC XENA (https://xena.ucsc.edu/). Additionally, microarray data from GSE14468, GSE37642-GPL96, GSE37642-GPL570, GSE12417_GPL96, and GSE10358, as well as RNA-seq data from GSE106291 and GSE165656, were obtained from the Gene Expression Omnibus (GEO, https://www.ncbi.nlm.nih.gov). All statistical analyses were performed using R (version 4.2.3). Kaplan-Meier analysis with log-rank tests was conducted to assess survival differences between groups using the "survminer" R package.

Molecular docking

Obtain the 3D structure of the protein (PDB ID: 6G6K, the crystal structure of the human MYC:MAX bHLHZip complex) from the RCSB database. Convert the ligand's SDF structure from the PubChem database to PDB format using OpenBabel. Utilize AutoDock Tools 1.5.6 for protein preparation, including removing water molecules, adding hydrogens, and converting both the protein and ligand into PDBQT format. Perform molecular docking simulations using AutoDock Vina. In the MYC-MAX crystal complex, residue numbering includes both MYC and MAX sequences. As a result, residue 938 in the complex corresponds to residue 406 in the MYC protein alone.

MD simulation

MD simulations were conducted using GROMACS 2020.3 software. The amber99sb-ildn force field and the general amber force field were used to generate the parameter and topology of proteins and ligands, respectively. The simulation box size was optimized with the distance between each atom of the protein and the box greater than 1.0 nm. Then, fill the box with water molecules based on a density of 1. To neutralize the system, Cl- and Na+ ions replaced the water molecules. Energy minimization was performed using the steepest descent method for 50,000 steps to reduce system energy and eliminate unreasonable contacts or atom overlaps. After energy minimization, the first-phase equilibration was done with the NVT ensemble at 300 K for 100 ps to stabilize the system's temperature. Second-phase equilibration was simulated with the NPT ensemble at 1 bar and 100 ps. The main goal of the simulation is to optimize interactions between the target protein, solvent, and ions for full pre-equilibration. All MD simulations were conducted for 50 ns at 300 K and 1 atmosphere in an isothermal-isobaric ensemble. Temperature and pressure were controlled by the V-rescale and Parrinello-Rahman methods with coupling constants of 0.1 ps and 0.5 ps, respectively. The Lennard-Jones function calculated the Van der Waals forces with a nonbond cutoff distance of 1.4 nm. Bond lengths were constrained using the LINCS algorithm. Long-range electrostatic interactions were calculated with the Particle Mesh-Ewald method using a Fourier spacing of 0.16 nm.

Lentivirus construction and infection

Lentiviral vectors for overexpressing RRP9 and MYC were GV492 (GeneChem, China) and GV703 (GeneChem, China), featuring the element sequences Ubi-MCS-3FLAG-

CBh-gcGFP-IRES-puromycin and CMV enhancer-MCS-3FLAG-EF1a-ZsGreen1-T2A-puromycin, respectively. The knockdown target sequences for RRP9 and MYC were GCTTGACCTTCTCTGTGACAT and GAATGTCAAGAGGCGAACACA, respectively. The lentiviral vector for both genes was GV493 (GeneChem, China) with the element sequence hU6-MCS-CBh-gcGFP-IRES-puromycin. For lentiviral packaging, 293T cells were co-transfected with a single plasmid or negative control siRNA, along with two helper plasmids as outlined above. The supernatant containing packaged lentivirus was harvested, concentrated via centrifugation, and purified by filtration. Subsequently, Kasumi-1 and MV4-11 cells were infected with these lentiviruses and the control lentiviruses.

mRNA stability assay

Cells in the logarithmic growth phase were seeded at a density of 2×10^5 cells/mL. Actinomycin D was added to a final concentration of 5 μg/mL, and samples were incubated for 0 h, 2 h, 6 h, and 8 h. At each time point, cells were harvested for qPCR analysis. The mRNA half-life was calculated by determining the ΔCt values, obtained by subtracting the average Ct value at 0 h from those at subsequent time points. The relative mRNA abundance was plotted using GraphPad Prism 8.0, and the mRNA decay rate was assessed through nonlinear regression curve fitting using a single-phase decay model.

ChIP assay

ChIP was performed using the SimpleChIP™ Enzymatic Chromatin IP Kit (9003S, Cell Signaling Technology, USA). MV4-11 cells (4×10^6 per ChIP) were cross-linked

with 1% formaldehyde for 10 minutes at room temperature, and the reaction was quenched with glycine at a final concentration of 0.125 M. Cells were collected by centrifugation and washed twice with ice-cold PBS. Micrococcal nuclease (0.5 μL per 4×10^6 cells) was added to digest DNA into fragments ranging from 150 to 900 bp. The nuclear membrane was disrupted by repeated sonication, and the supernatant, containing cross-linked chromatin, was collected after centrifugation. Chromatin concentration and digestion efficiency were evaluated following the manufacturer's instructions. ChIP was performed overnight at 4°C using anti-H3 (4620S), anti-c-MYC (9402S) antibodies, or normal IgG control (2729S, Cell Signaling Technology, USA). Input DNA, isolated from sonicated lysates prior to immunoprecipitation, served as a positive control. Immune complexes were captured using Protein G Magnetic Beads (Cell Signaling Technology, USA). Cross-links were reversed by incubating the samples at 65°C for 2 hours. The resulting DNA was purified using a spin column and subjected to ChIP-PCR analysis. Primer sequences targeting the c-MYC binding site on the RRP9 promoter are listed in Table S6.

RNA gel electrophoresis

Prepare a 1% agarose gel containing GelRed. Mix 1 μg of total RNA sample with an equal volume of RNA Loading Dye, then subject the mixture to electrophoresis at 120V for approximately 30 minutes. Visualize the RNA bands using a UV gel imaging system.

Electron microscopy observation of nucleoli

Treated cells were fixed in 0.5% glutaraldehyde (Beyotime, China) for 10 minutes at 4°C, followed by fixation in 2.5% glutaraldehyde (LEAGENE, China). Cells were then

post-fixed in 1% osmium tetroxide, dehydrated in acetone, and infiltrated with Epox 812 resin before embedding. Semithin sections were stained with methylene blue, while ultrathin sections were cut using a diamond knife and stained with uranyl acetate and lead citrate. Sections were examined using a JEM-1400FLASH Transmission Electron Microscope (JEOL, Japan).

Immunofluorescence

Treated cells were fixed with 4% paraformaldehyde, permeabilized with 0.2% Triton X-100, and blocked with 5% BSA. Cells were incubated overnight at 4°C with anti-NPM1 antibody (92825T, Cell Signaling Technology, USA) diluted in 1% BSA/PBS, followed by fluorophore-conjugated secondary antibody for 1 hour at room temperature in the dark. Nuclei were stained with DAPI and mounted with antifade medium. Whole-slide fluorescent images were acquired using the Pannoramic SCAN II scanner (3DHISTECH Kft) and analyzed with CaseViewer software. Fluorescence intensity was quantified using ImageJ.

Polysome fractionation assay

In summary, it involved preparing a sucrose gradient with a gradient former (Biocomp, Canada), separating and precipitating polysomes using an ultra-high-speed centrifuge (Beckman, USA), and performing polysome fractionation with a fully automated density gradient preparation and separation system (Biocomp, Canada). Finally, RNA was extracted using the Trizol method.

Nascent protein detection assays

Nascent protein detection assays were performed using the Click-iT® Plus OPP Protein Synthesis Assay Kit (Thermo Fisher Scientific, USA) in accordance with the manufacturer's protocol. Cells were incubated in complete 1640 medium supplemented with 20 μM Click-iT® OPP for 30 minutes. Following OPP labeling, cells were fixed with 3.7% formaldehyde for 15 minutes, permeabilized with 0.5% Triton X-100 for 15 minutes and stained with Alexa Fluor® 594 for 30 minutes at room temperature. After staining, cells were washed thoroughly with Click-iT® reaction rinse buffer and analyzed using the FACSymphony™ A5 Flow Cytometer (BD, USA).

Supplementary Tables

Table S1. The half-maximal inhibitory concentrations of chidamide and cytarabine on AML cell lines

	time (h)	MV4-11(μM)	Kasumi-1(μM)
chidamide	24	3.576	3.225
	48	2.830	0.899
	72	0.272	0.243
cytarabine	24	>20	>20
	48	3.061	4.756
	72	1.011	0.718

Table S2. Clinical information of the six patients with AML

N0.	Age/Sex	Disease status	WBC (10^9/L)	Karyotype	Molecular features	LDH(IU/L)
1	65/M	t-AML	4.09	-4, -7, -9, del(9q), -13, -16, -17. etc.	*TP53*^{mut}, *CSF3R*^{mut}	533
2	67/F	relapsed	2.23	46, XX, [20]	*MLL-AF9*$^{+}$	234
3	54/F	refractory	7.43	46, XX, del (17) (p13) [15]/46. idem, +13, -21[5]	-	149
4	42/F	primary	8.49	46, XX [20]	*MLL-AF9*$^{+}$, *WT1*$^{+}$	202
5	67/M	relapsed	55.53	46, XY, t (11;19) (q23; p13.1) [20]	*MLL-ELL*$^{+}$, *NRAS* ^{mut}, *TET2*^{mut}, *STAG2*^{mut}	890
6	41/F	primary	4.98	46, XX [20]	*MLL-AF6*$^{+}$, *WT1*$^{+}$, *EV11*$^{+}$, *KRAS*^{mut}, *DNMT3A*^{mut}	147

M, male; F, female; t-AML, treatment-related acute myeloid leukemia; mut, mutation; +, positive.

Table S3. Clinical information for patients with AML who achieved complete remission using chidamide-based chemotherapy

N0.	Age/Sex	Disease status	Karyotype	Molecular features	Induction therapy	Response
1	31/F	primary	46, XX, t (6;11) (q27; q23) [20]	*MLL-AF6*$^{+}$, *EV11*$^{+}$	IA+ Chi	CR, MRD-
2	30/F	primary	46, XX, t (16;21) (p11; q22) [18]/46, XX [2]	*TLS/ERG*$^{+}$, *PTPN11*^{mut}, *KMT2C*^{mut}, *RUNX1*^{mut}, *SF1*^{mut}	IA+ Chi	CR, MRD+
3	47/M	r/r	46, XY, inv (16) (p13; q22) [20]	*CBF β/MYH11*$^{+}$, *FLT-TKD* ^{mut}, *Kit(D86)* ^{mut}	HAA+ Chi	CR, MRD-
4	41/F	r/r	46, XX, t (9;11) (p22; q23) [20]	*MLL-AF9*$^{+}$, *CEBPA*^{mut}, *TET2*^{mut}, *ASXL1*^{mut}, *WT1*$^{+}$	CAG+ Chi	CR, MRD-
5	14/F	CR，MRD+	45, X, -X, t (8;21) (q22; q23) [20]	*AML-ETO*$^{+}$, *JAK2*^{mut}, *PHF6*^{mut}, *KRAS*^{mut}	HD-AraC+Chi	CR, MRD-

F, female; IA, idarubicin and cytarabine; Chi, chidamide; CR, complete remission; MRD, minimal residual disease; mut, mutation; M, male; r/r, relapsed or refractory; HAA, homoharringtonine, aclarubicin, and cytarabine; CAG, cytarabine, aclarubicin, and granulocyte colony-stimulating factor; HD-AraC, High-dose cytarabine.

Table S4. Analysis of protein ligand molecular mechanics/Poisson–Boltzmann surface area

Energy	chidamide–MYC–MAX	cytarabine–chidamide–MYC–MAX
Van der Waals Energy (KJ/mol)	-221.843	-340.618
Electrostatic energy (kJ/mol)	-229.009	-191.361
Polar solvation energy (KJ/mol)	303.881	345.651
Nonpolar solvation Energy (KJ/mol)	-17.787	-75.745
Total Binding Energy (KJ/mol)	-164.758	-262.073
TΔS(KJ/mol)	23.086	27.454
Total Binding Free Energy (KJ/mol)	-141.672	-234.619

Table S5. Sequencing-Coverage-and-Quality-Statistics

Sample ID	Total number of sequenced reads	Total number of uniquely mapped reads	RNA integrity number (RIN)	Ratio of all reads aligned to rRNA regions to total uniquely mapped reads (rRNA rate)	Ratio of exon-mapped reads to total uniquely mapped reads (Expression Profile Efficiency)	Total number of detected transcripts with reads ≥1
CTR1	54538434	51098405	9.4	0.90%	90.24%	78637
CTR2	45551326	42908446	9.7	0.83%	90.40%	76602
CTR3	39616008	37295295	9.8	0.83%	90.84%	71573
CH1	43829082	41277548	9.8	0.80%	89.98%	82002
CH2	41172530	38739946	9.8	0.90%	89.82%	78912
CH3	45219076	42514066	9.8	0.93%	89.15%	79580
Ara1	44640322	41922316	9.8	0.85%	89.26%	75621
Ara2	46488750	43739325	8.9	0.89%	89.14%	78256
Ara3	46730236	43976248	9.7	0.75%	89.54%	76160
C_A1	45732802	42938819	9.9	1.02%	88.88%	85068
C_A2	50588478	47435717	9.2	1.05%	88.47%	82307
C_A3	39236104	36947910	9.2	1%	87.67%	79753

Table S6. Primer sequences for qPCR (5' to 3')

Oligo name	Primers sequence（5' to 3'）
5.8S F	ACTCGGCTCGTGCGTC
5.8S R	GCGACGCTCAGACAGG
18S F	GGGGCCCGAAGCGTTTACTTTG
18S R	CAAGAATTTCACCTCTAGCGGCGC
28S F	AGAGGTAAACGGGTGGGGTC
28S R	GGGGTCGGGAGGAACGG
RRP9-promoter F	GTGGACACTCGATCTGGCAT
RRP9-promoter R	GGGAGGCTGCGTCTGTATC
NOP16 F	GGATCCGCGATGTCTCTCTTTG
NOP16 R	CCTCCAGGTCATTCAGCACATAG
NOP56 F	TCCCGTGCCAAAGTTAAGTTTAA
NOP56 R	GTGGCATTGTCGTTGATGATCTT
DDX21 F	TGAAGCTGCCAGTGAAGAAAGTA
DDX21 R	TCAAAGGGATGGCAAAGGAGAAT
GAPDH F	CGCTGAGTACGTCGTGGAGTC
GAPDH R	TGATCTTGAGGCTGTTGTC

Supplementary Figures

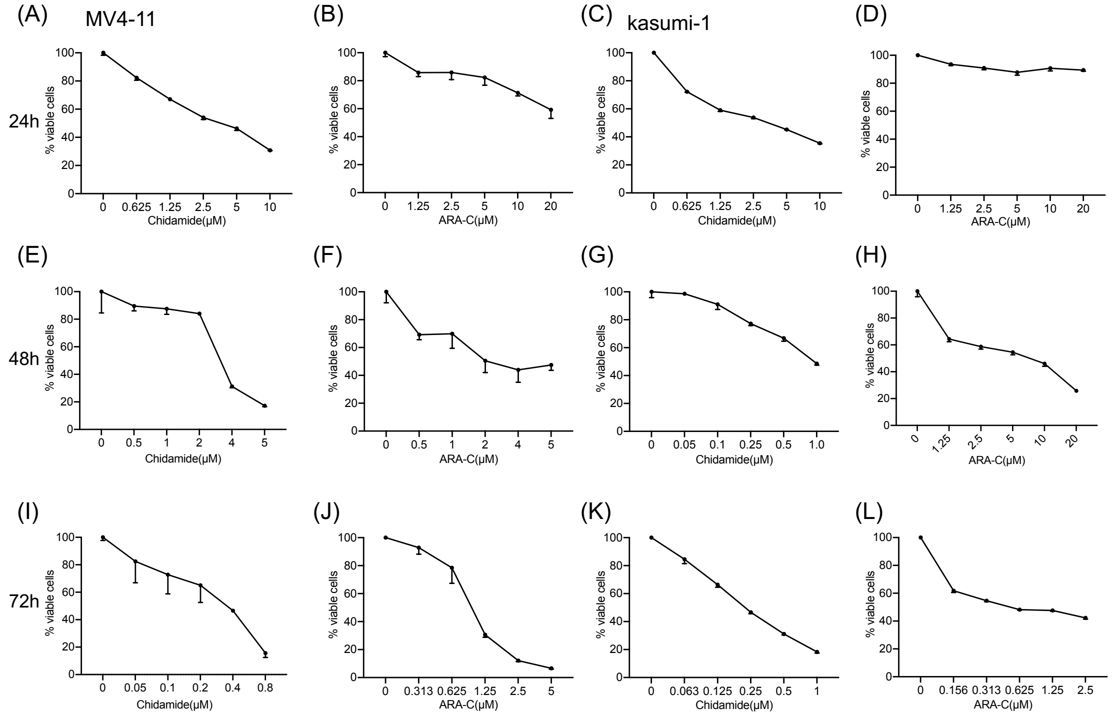

FIGURE S1. Chidamide and cytarabine induce AML cell death in a dose- and time-dependent manner.

(**A–L**), MTT assay assessing the cytotoxic effect of AML cells treated with varying concentrations of chidamide or cytarabine for 24 hours (**A–D**), 48 hours (**E–H**), and 72 hours (**I–L**).

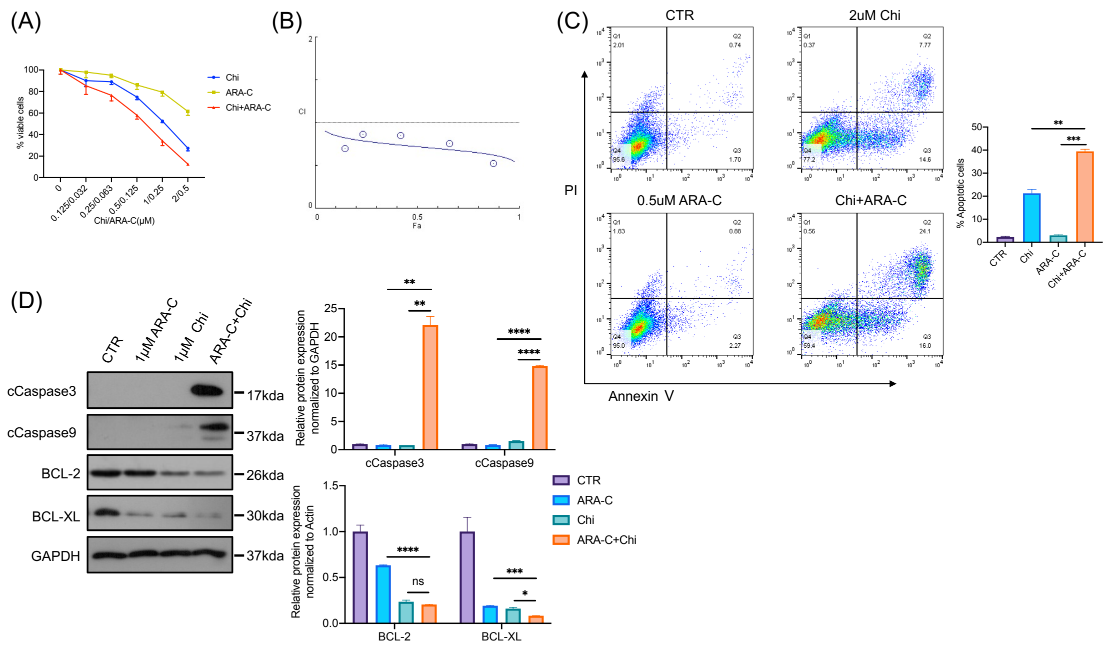

FIGURE S2. Synergistic therapeutic effects of chidamide and cytarabine in HL60 cells.

(A), Cytotoxic effects of chidamide and cytarabine, alone or in combination on HL60 cells. (B), Combination index plot, the horizontal axis (Fa) represents the inhibition rate, while the vertical axis shows CI values. (C), Significant differences were observed between chidamide and combination (*P*=0.0051, unpaired t-test) and between cytarabine and combination (*P*=0.0003, unpaired t-test). (D), Western blot analysis of cleaved caspase-3, cleaved caspase-9, BCL-2, and BCL-XL in HL60 cells. ARA-C vs. combination (*P*=0.0024, *P*<0.0001, *P*<0.0001, 0.0006). Chidamide vs. combination (*P*=0.0024, *P*<0.0001, ns, 0.0150) (unpaired t-test). CTR (control), Chi (chidamide), ARA-C (cytarabine), Chi+ARA-C (chidamide + cytarabine); cCASP3, cleaved Caspase-3; cCASP9, cleaved Caspase-9, ns: No statistical significance, *P < 0.05, **P < 0.01, ***P < 0.001, ****P < 0.0001.

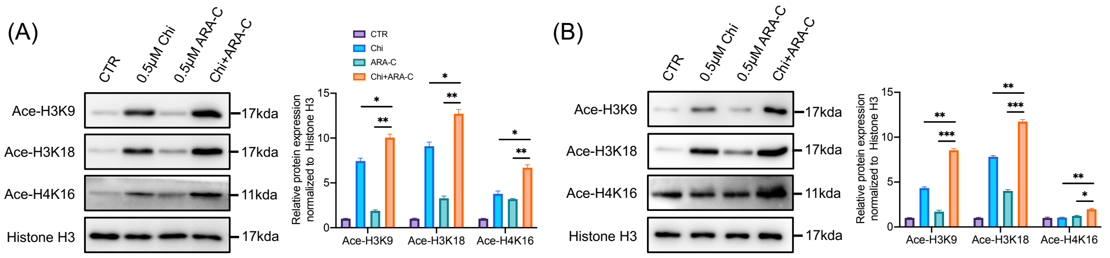

FIGURE S3. Chidamide and cytarabine synergistically up-regulate histone acetylation levels in AML cells.

(A, B), Western blot analysis showed that the combination of chidamide and cytarabine significantly increased acetylation of H3K9, H3K18, and H4K16 in MV4-11 (left) and Kasumi-1 (right) cells. In MV4-11 cells, compared to chidamide alone, the combination showed higher acetylation levels (H3K9: P= 0.0180; H3K18: P = 0.0166; H4K16: P= 0.0113), and compared to cytarabine alone (H3K9: P=0.0012; H3K18: P= 0.0016; H4K16: P=0.0043). In Kasumi-1 cells, similar increases were observed compared to chidamide (P=0.0014, 0.0021, 0.0068) and cytarabine (P= 0.0006, 0.0006, 0.0129) for H3K9, H3K18, and H4K16, respectively (unpaired t-test). *$P < 0.05$, **$P < 0.01$, ***$P < 0.001$.

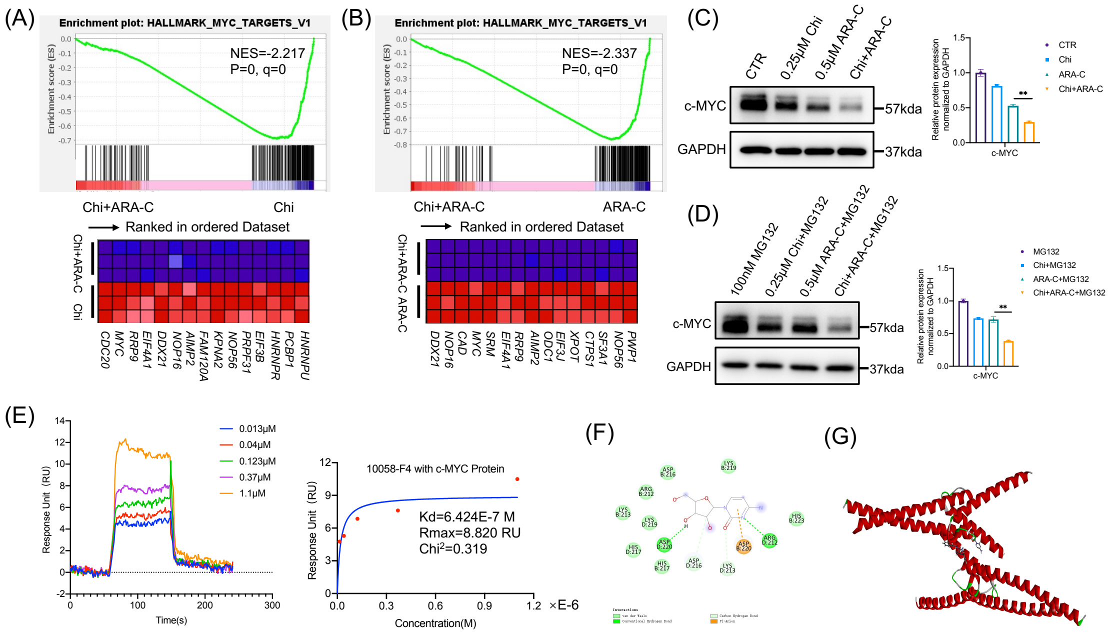

FIGURE S4. Chidamide combined with cytarabine synergistically suppresses the MYC signaling pathway

(**A, B**), The combination of chidamide and cytarabine significantly suppressed the MYC pathway compared to chidamide alone (left) and to cytarabine alone (right). (**C**), Effects of chidamide and cytarabine on c-MYC protein levels in HL60 cells, with quantification of the results. Significant difference between cytarabine and combination (P=0.0052, unpaired t-test). (**D**), c-MYC protein levels in HL60 cells were evaluated and quantified after treatment with chidamide and cytarabine in the presence of MG132. Significant difference between cytarabine and combination (P=0.0080, unpaired t-test). (**E**), SPR analysis shows concentration-dependent binding of 10058-F4 to c-MYC, meeting quality control criteria. (**F, G**), Two-dimensional (left) and three-dimensional (right) molecular docking of cytarabine with the chidamide–MYC–MAX complex. CTR (control), Chi (chidamide), ARA-C (cytarabine), Chi+ARA-C (chidamide + cytarabine). **P < 0.01.

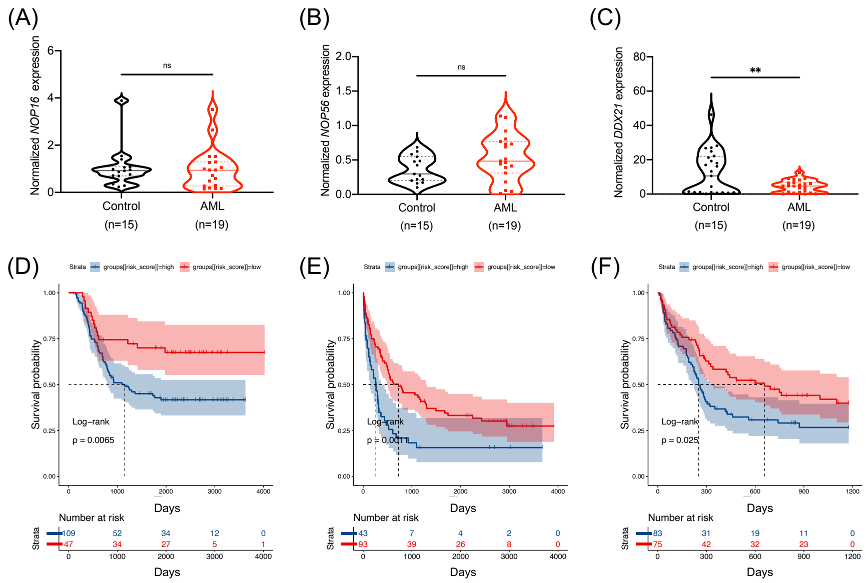

FIGURE S5. Elevated RRP9 expression is associated with poor prognosis in AML. (**A–C**) *NOP16*, *NOP56*, and *DDX21* mRNA levels in PBMCs from AML patients and healthy controls (*NOP16* and *NOP56*, ns; *DDX21*, *P*=0.0074; unpaired t-test). (**D**), Kaplan-Meier analysis of TARGET-AML RNA-seq data showed a significant correlation between RRP9 expression and prognosis (*P*=0.0065, Kaplan Meier analysis). (**E, F**), Kaplan-Meier analysis of microarray datasets, GSE37642_GPL570 (*P*=0.0011, Kaplan Meier analysis) and GSE12417_GPL96 (*P*=0.025, Kaplan Meier analysis), also revealed significant associations between RRP9 expression and patient prognosis. ***P*<0.01.

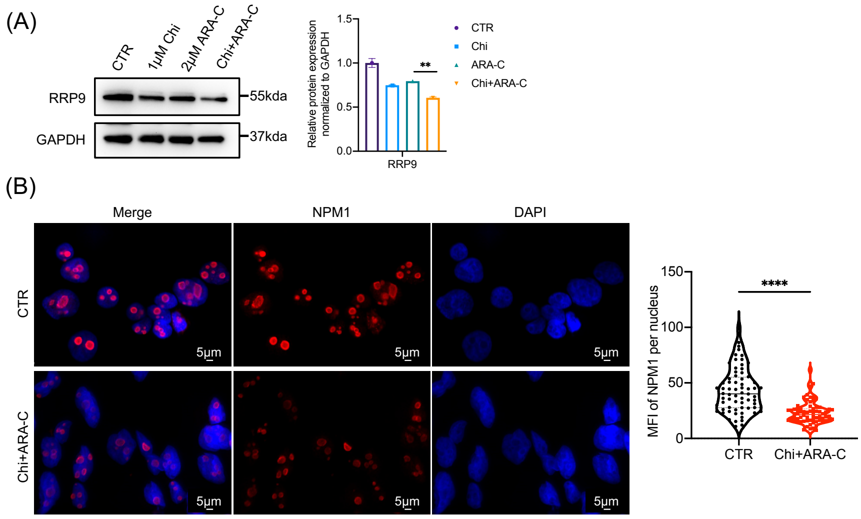

FIGURE S6. Chidamide combined with cytarabine inhibits ribosome biogenesis in AML cells by downregulating RRP9.

(**A**)**,** Effects of chidamide and cytarabine on RRP9 protein levels in HL60, showing a significant difference between cytarabine monotherapy and the combination treatment (*P*=0.0014, unpaired t-test). (**B**), Immunofluorescence analysis showed that the fluorescence intensity of NPM1 in HL60 cells was significantly decreased after treatment with chidamide and cytarabine (*P*<0.0001, unpaired t-test) (n=68). CTR (control), Chi+ARA-C (chidamide + cytarabine). MFI: Mean fluorescence intensity. ***P* < 0.01, *****P* < 0.0001.
